# Plants, Cells, Algae, and Cyanobacteria In Vitro and Cryobank Collections at the Institute of Plant Physiology, Russian Academy of Sciences—A Platform for Research and Production Center

**DOI:** 10.3390/biology12060838

**Published:** 2023-06-09

**Authors:** Natalya Yuorieva, Maria Sinetova, Ekaterina Messineva, Irina Kulichenko, Artem Fomenkov, Olga Vysotskaya, Ekaterina Osipova, Angela Baikalova, Olga Prudnikova, Maria Titova, Alexander V. Nosov, Elena Popova

**Affiliations:** K.A. Timiryazev Institute of Plant Physiology of Russian Academy of Sciences, Botanicheskaya 35, 127276 Moscow, Russia; yuorieva@mail.ru (N.Y.); maria.sinetova@mail.ru (M.S.); titomirez@mail.ru (M.T.); alexv.nosov@mail.ru (A.V.N.)

**Keywords:** plant biotechnology, in vitro collection, plant germplasm collection, microalgae, cyanobacteria, plant cells, meristem, cryobank, cryopreservation, plant cell culture, transgenic potato

## Abstract

**Simple Summary:**

This review highlights six genetic collections of algae, cyanobacteria, and plant materials maintained at the Institute of Plant Physiology of the Russian Academy of Sciences (IPPRAS) since the 1950–1970s using in vitro and cryopreservation techniques. The in vitro collections conserve over 430 strains of algae and cyanobacteria, more than 200 transgenic and non-transgenic potato clones, 117 cell cultures, and 50 strains of hairy and adventitious root cultures of medicinal and model plant species. The IPPRAS cryobank of plant genetic resources preserves in vitro-derived germplasm and seeds of wild and cultivated plants from 457 species and 74 families. This review discusses the collections’ major activities and their extensive use in research, biotechnological interventions, commercial application, and biodiversity conservation. We also emphasize the role of in vitro collections as a genetic basis for green biotechnologies.

**Abstract:**

Ex situ collections of algae, cyanobacteria, and plant materials (cell cultures, hairy and adventitious root cultures, shoots, etc.) maintained in vitro or in liquid nitrogen (−196 °C, LN) are valuable sources of strains with unique ecological and biotechnological traits. Such collections play a vital role in bioresource conservation, science, and industry development but are rarely covered in publications. Here, we provide an overview of five genetic collections maintained at the Institute of Plant Physiology of the Russian Academy of Sciences (IPPRAS) since the 1950–1970s using in vitro and cryopreservation approaches. These collections represent different levels of plant organization, from individual cells (cell culture collection) to organs (hairy and adventitious root cultures, shoot apices) to in vitro plants. The total collection holdings comprise more than 430 strains of algae and cyanobacteria, over 200 potato clones, 117 cell cultures, and 50 strains of hairy and adventitious root cultures of medicinal and model plant species. The IPPRAS plant cryobank preserves in LN over 1000 specimens of in vitro cultures and seeds of wild and cultivated plants belonging to 457 species and 74 families. Several algae and plant cell culture strains have been adapted for cultivation in bioreactors from laboratory (5–20-L) to pilot (75-L) to semi-industrial (150–630-L) scale for the production of biomass with high nutritive or pharmacological value. Some of the strains with proven biological activities are currently used to produce cosmetics and food supplements. Here, we provide an overview of the current collections’ composition and major activities, their use in research, biotechnology, and commercial application. We also highlight the most interesting studies performed with collection strains and discuss strategies for the collections’ future development and exploitation in view of current trends in biotechnology and genetic resources conservation.

## 1. Introduction

Conservation of plant biodiversity was first articulated in the early 20th century and has since become a priority of national policies and international treaties [1]. Genetic resources of staple crops and their wild relatives conserved ex situ in genebanks worldwide underpin the research and breeding programs that provide researchers and farmers access to diverse and well-documented high-quality plant materials [2,3,4]. The Second Report on the State of the World’s Plant Genetic Resources for Food and Agriculture indicated that about 7.4 million accessions of crops are maintained ex situ in more than 1750 genebanks [5]. However, genetic erosion and the risks to crop diversity imposed by climate change are not the only concerns of the scientific community. Human life largely depends on the medicines and fuel provided by wild plants [6,7], but their diversity is also depleting rapidly [8]. Of the 25,791 non-hybrid plant species with documented medicinal use, 5411 (21%) are represented by assessments on the global International Union for Conservation of Nature (IUCN) Red List of Threatened Species [7,9]. According to another rough estimate, about 15,000 medicinal plant species are threatened with extinction from overharvesting and habitat destruction [10]. Only a small portion of these species is represented in the genebanks. Sustainable production of novel food and plant-derived bioactive components through biotechnological approaches such as in vitro plant propagation or cell farming could eliminate or significantly reduce the industry’s dependence on wild plant resources [11,12,13]. However, to be economically effective, these technologies require a large genetic base of the appropriate starting material, prone to further trait-based selection [14]. Unlike crop genebanks and botanic gardens, little is known about genetic collections of biotechnologically valuable plant materials in vitro, e.g., plant cell and hairy root cultures, embryogenic cell lines, in vitro clones with superior qualities, etc. Information about some of these collections can be driven from publications [15,16,17] or open online resources, while others belong to commercial companies and are protected by patents and commercial confidentiality laws.

Microalgae and cyanobacteria are widely used as model organisms in plant physiological and biochemical studies due to their simple organization, rapid growth, and moderate nutritional requirements [18]. High biomass productivity; metabolic plasticity; and the ability to produce large amounts of proteins, lipids, polysaccharides, carotenoids, and vitamins make these organisms attractive for applications in biotechnology [19,20]. Furthermore, culture collections of microalgae and cyanobacteria enable the sustainable use of their diversity for fundamental and applied research. Algae collections ensure the long-term conservation and distribution of strains with a known taxonomic identification, provide information for their successful cultivation, and offer a variety of services to the industrial, scientific, and educational communities [18].

Most of in vitro collections are maintained by regular transfers of living material to a fresh medium, which is laborious, time-consuming, and expensive. Not surprisingly, in vitro collections are often highly specialized, focusing on certain taxa or material types, e.g., specific crops, embryogenic cell lines for forestry improvement programs, or cultures of undifferentiated plant cells [16,21,22,23].

To reduce the maintenance cost and mitigate the risks of (epi)genetic variations and human errors associated with long-term in vitro maintenance, cryogenic (−196 °C) storage has been successfully applied to some plant in vitro collections of high agricultural and horticultural importance [21,22,23,24,25,26,27]. Likewise, cold storage (−80–+4 °C) and cryopreservation have been implemented to conserve the diversity of biotechnological materials, including plant cell cultures, adventitious and hairy root cultures, and algae, although large-scale low-temperature conservation is still limited to few examples [28,29,30,31].

The K.A. Timiryazev Institute of Plant Physiology of the Russian Academy of Sciences (IPPRAS) is a research institute with two large biotechnology departments holding unique bioreactor systems for the cultivation of plant suspension cell cultures and algae from the laboratory (5–20 L) to the pilot (75 L) and the semi-industrial (150–630 L) scale. The institute also hosts eight plant material and algae collections, some of them are of national importance:

- Culture collection of microalgae and cyanobacteria IPPAS.
- In vitro collection of transgenic potato plants.
- All-Russian collection of plant cell cultures.
- Hairy root culture collection.
- Adventitious root culture collection.
- IPPRAS cryobank.
- *Vitis* spp. plant collection.
- Collection of succulent plants.

**Figure 1:**
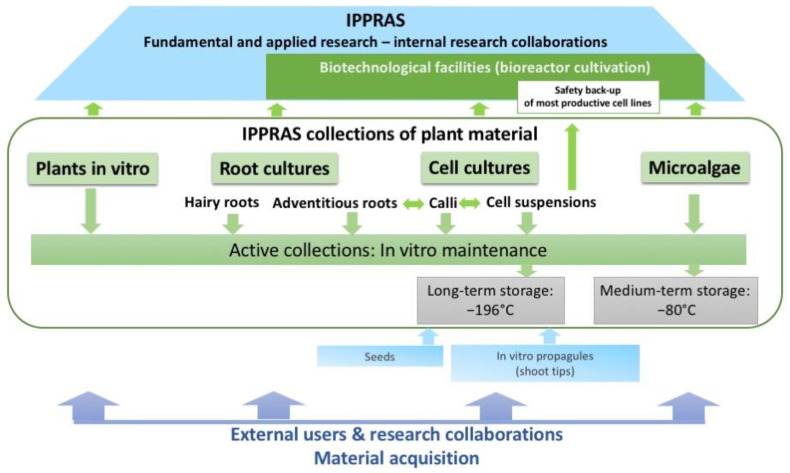
Schematic representation of the collections of higher plant material, algae, and cyanobacteria at IPPRAS, storage methods, and interaction with IPPRAS biotechnological facilities, internal collaborators and external users.

Most collections implement in vitro cultivation as the main method for routine maintenance (Figure 1). Collections of succulents and *Vitis* spp. are maintained mostly as greenhouse plants, and are not covered in this review.

The in vitro and cryobank collections, except for adventitious root cultures, were initiated between the periods of the 1950s and the 1970s, and are now the oldest and most diverse collections of their kind in the country, with total holdings of in vitro cultured materials above 780 strains/clones and over 1000 cryopreserved seed specimens (Appendix A, Table A1). These collections represent different levels of plant organization, from individual cells (cell culture collection) to organs (hairy and adventitious root cultures, shoot apices, seeds) to in vitro plants (Appendix A, Table A1). Collectively, the diversity of the materials conserved, the close interaction with biotechnological facilities (Figure 1), and the expertise and research data acquired over time create an appealing case for the successful conservation of different bioresource types “under one roof”, which has never been covered in a publication. Here, we provide a comprehensive review of the current collections’ composition and their use in research and biotechnological programs inside and outside the host institute. We highlight the most interesting studies performed using collection strains and discuss potential directions for future collection development and exploitation.

## 2. All-Russian Collection of Plant Cell Cultures

### 2.1. Plant Cell Culture Collections around the World

Plant cell culture is a unique, artificially created in vitro biological system—a population of constantly proliferating undifferentiated plant cells. Cell cultures maintained on the surface of the solid nutrient medium (callus) or in a liquid medium (cell suspension) often retain the ability of the donor plant to produce specific secondary metabolites of high pharmacological value [14,32]. Rapid growth under sterile controlled conditions and stable biosynthesis of the desired compounds make the cell cultures an attractive alternative to wild and plantation-grown medicinal plants for biomass and phytochemical production [33,34]. Cell cultures lacking organismic controls can also be used as model systems in physiological, biochemical, and molecular studies, i.e., investigating the regulation of cell growth and secondary metabolite biosynthesis, stress signaling, and stress tolerance [33].

Pilot production projects using plant cell cultures were developed in the 1980s–1990s (e.g., [35,36,37,38,39,40,41]). Regrettably, most of them were later closed, facing constraints of high production costs and low content of the desired phytochemicals making such hi-tech production unprofitable or uncompetitive [16,42]. However, due to recent trends toward sustainable and eco-friendly production processes, plant cell cultures are retrieving increasing attention and a new spin [16]. Recent reviews [43,44,45,46] highlighted over 20 companies using plant cell culture-derived substances in their cosmetic products. The concept of cell culture-produced biomass as a component of food supplements was also revived [47,48,49]. Recent studies demonstrated that teupolioside, a biologically active phenylpropanoid glycoside produced from the cell culture of *Ajuga reptans* L., was effective for wound healing and exhibited anti-inflammatory activity in the model of induced colitis [50,51]. Cell culture extracts containing different concentrations of teupolioside have been certified as food supplement ingredients in Europe [52]. Additionally, the commercial production of the anti-cancer drug Taxol© (a trading name of paclitaxel) from the cell cultures of *Taxus* spp. has been known for a long time [14,53].

The induction and maintenance of the cell strains with intensive growth accompanied by high and stable metabolite production is a prerequisite for successful cell-based biotechnology. However, the collections of cell cultures are few and mainly limited to developed countries. The largest and most online visible collections are the Plant Cell Culture Library of the University of Massachusetts Amherst (>1000 plant species, USA, https://www.umass.edu/ials/pccl-database, accessed on 23 March 2023); the cell culture collection of the Leibniz Institute DSMZ-German Collection of Microorganisms and Cell Cultures (>80 families, last mentioned in [16]); the VTT Culture Collection; collections from Finland (23 species, [54]); RIKEN BRC Plant Cultured Cell Resources, Japan (32 species, [55]); the cell culture collection of the University of Debrecen, Hungary [15]; and the collection of in vitro plant cell cultures at the Institute of Experimental Botany (Czech Republic, >20 species, [56]). These collections hold cell strains with anti-cancer, antimicrobial, antioxidant, insecticidal, and other properties as well as model cell strains of tobacco, *Arabidopsis thaliana* (L.) Heynh. (wild-type and mutants), and plant species with sequenced genomes.

The All-Russian Collection of Plant Cell Cultures hosted by IPPRAS is the oldest and most diverse Russian collection of plant cell cultures [57], with a mandate to receive and deposit cell strains from other institutions for patent purposes.

### 2.2. All-Russian Collection of Plant Cell Cultures—Historical Perspective and Current Composition

**Figure 2:**
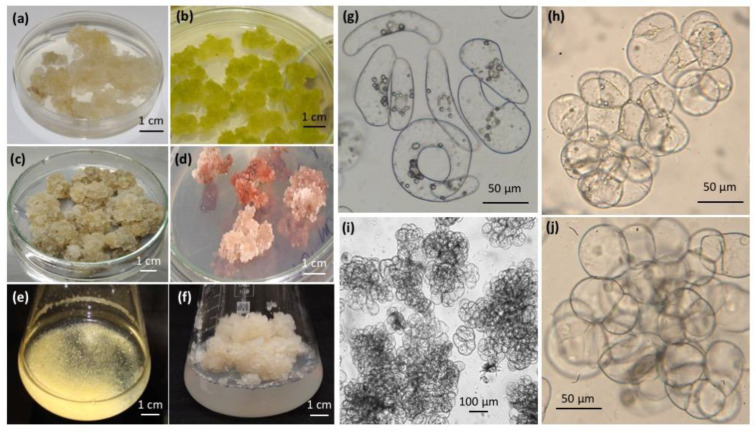
Examples of the cell strains maintained in the All-Russian Collection of Plant Cell Cultures: callus cultures of (**a**) *Polyscias fruticosa*; (**b**) *Panax japonicus*; (**c**) *Ajuga turkestanica*; (**d**) *Ajuga reptans*. (**e**) Suspension cell culture of *Polyscial fruticosa*. (**f**) 64-year-old callus culture of *Panax ginseng*. (**g**–**j**) Suspension cell cultures under a microscope for the following species: (**g**) *Alcea kusariensis*; (**h**) *Ajuga reptans*; (**i**) *Polyscias filicifolia*; and (**j**) *Nicotiana tabaccum*. Photographs © IPPRAS.

**Figure 3:**
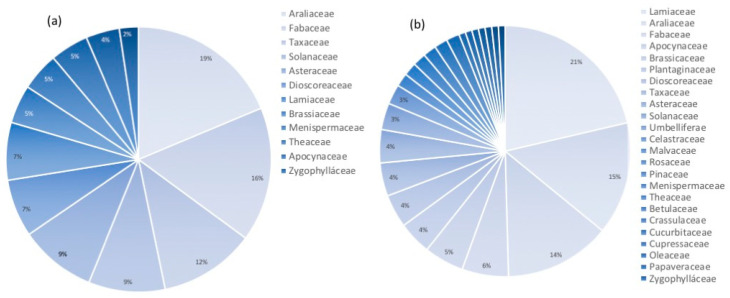
Plant families represented in the All-Russian Collection of Plant Cell Cultures (modified from [30]): (**a**) core collection (43 cell culture strains); (**b**) core collection plus experimental cell culture strains (117 strains in total). Core collection is composed of comprehensively researched strains with optimized culture conditions and passport data (growth, cytological, biochemical characteristics, etc.) recorded. For (**b**) percentages below 3% are not labeled in the chart.

The first cell cultures in Russia were developed by Prof. Raisa G. Butenko and her research team at IPPRAS in the late 1950s–mid 1960s [30]. These included cell cultures of *Panax ginseng* C. A. Mey., *Rauvolfia serpentina* Benth. ex Kurz, *Dichroa febrifuga* (Lour.) Y. De Smet and C. Granados, *Catharanthus roseus* (L.) G. Don, *Dioscorea deltoidea* Wall., and other medicinal plant species [58,59]. Some of those cultures are still maintained in the active collection by periodic subcultures. The collection composition and use have been recently reviewed [30]. Historically, the collection has been focused on developing and maintaining cell strains accumulating isoprenoid compounds (furostanol glycosides, ginsenosides, taxoids, etc.) [60], although model strains of *Nicotiana tabaccum* L. and *Arabidopsis thaliana* are also present (Figure 2) [30]. As of March 2023, the collection holds 43 cell culture strains of 24 plant species as the core collection (Figure 3a). Furthermore, 74 strains of 32 plant species are cultured for experimental purposes (Figure 3b). The most represented families are Araliaceae, Fabaceae, and Lamiaceae. The core collection is mostly formed by cell strains producing high quantities of secondary metabolites valuable for human health. These strains have optimized culture conditions and the passport data (growth, cytological, biochemical characteristics, etc.) recorded. The core collection includes, for example, cell culture strains of *Dioscorea deltoidea*, with total content of protodioscin, deltoside and their 25(*S*)-isomers up to 4.6–5.7% of the dry cell weight (DW); *Panax ginseng* and *Panax japonicus* (T. Nees) C.A. Mey. cell strains (ginsenoside and their derivatives up to 3.5% DW); *Tribulus terrestris* L. (total content of furostanol glycosides 0.1% DW); *Polyscias filicifolia* L. H. Bailey and *P. fruticosa* Harms (total content of polysciosides and their derivatives 0.5–3.0% DW). The cell cultures of some species, e.g., *Dioscorea deltoidea*, *Mandragora officinarum* L., and *Medicago sativa* L., have been maintained by periodic subcultures since the 1970s or 1980s. The experimental collection contains recently acquired cell strains at different stages of growth optimization, biochemical evaluation, and screening for biological activities. These include, for example, cell cultures of medicinal plants *Sutherlandia frutescens* (L.) W. T. Aiton, *Ajuga turkestanica* (Regel) Briq., *Alhagi maurorum* Medik., *Maackia amurensis* Rupr., *Cladochaeta candidissima* DC., *Alcea kusariensis* (Iljin ex Grossh.) Iljin, and *Panax vietnamensis* Ha and Grushv.

### 2.3. Using Plant Cell Culture Strains in the Research

Cell culture strains from the collection have been extensively used as models to study plant cell growth and biosynthesis regulation in isolated cells compared to organized tissues or whole plants. For example, wild-type and mutant cell strains of *Arabidopsis thaliana* were used to study the interaction of ethylene and abscisic acid signaling pathways [61], sodium ion intake and transport [62,63], nitric oxide effects [64], as well as the regulation of zinc homeostasis genes in plant cells [65].

The variety of cell lines developed from different species belonging to the same family (Araliaceae, Fabaceae, Taxaceae) allows the investigation of taxon-related variations in primary and secondary metabolism in the cell cultures. In addition, cell strains developed from different plant parts (explants) or donor plants from different geographical locations and maintained on nutrient media with varied mineral and phytohormonal composition are excellent models to study the intra-specific variations in cell culture properties and the role of explant source and cultivation conditions on cell growth and biosynthesis. Recent studies performed on callus and suspension cell cultures of three yew species (*Taxus baccata* Thunb., *T. canadensis* Marshall, and *T. wallichiana* Zucc.) and two Taxus × media Rehder hybrids originating from different explants and grown in over 20 nutrient media revealed that genotype (the individual plant used for culture induction) was the most significant factor influencing the content and composition of taxoid compounds in cell biomass followed by species and medium formulation [66]. Two callus and two suspension cell lines of *Sutherlandia frutescens* induced from hypocotyl and cotyledon explants had distinct cell morphology but very similar profiles of secondary metabolites that differed from secondary metabolite composition in plant leaves [67]. By contrast, the content of fatty acids (FAs), primarily linoleic and linolenic, in cell cultures was influenced by both the explant origin and growth conditions (light or dark) [67]. In *Alhagi maurorum*, the explant type significantly affected the callus induction rate; in vitro seedlings were a superior explant source compared to ex vitro plants [68].

The stability of growth and biosynthetic characteristics of the cell cultures over time is one of the main questions of interest in biotechnological collections and production companies. The cell culture collection at IPPRAS, with its long-term cultured strains, is well-positioned to be utilized in experiments exploring stability monitoring in cell cultures of different taxa. For example, the cell suspension of *Panax japonicus* maintained by periodic subcultures for over 20 years fully retained its growth characteristics [30] and produced a broad spectrum of ginsenosides, including protopanaxatriols (Re, Rg_1_, Rf), protopanaxadiols (Rb_1_, Rb_2_, Rc, Rd), ginsenoside R_0_, and malonyl-ginsenosides [69]. The main growth parameters recorded during cultivation in the 20-L, 75-L, and 630-L bioreactors remained unchanged for the suspension cell culture of *Polyscias filicifolia* after five years of maintenance by periodic subcultures [70].

### 2.4. Biotechnological Application of Plant Cell Strains from the Collection

Before being used in biotechnology, cell strains are assessed following a standard evaluation scheme which includes cytological analysis (cell size, form, level of aggregation), reference photographs, evaluation of growth characteristics (growth curves, growth index, specific growth rate, productivity, and economic coefficient as described in Appendix A, Table A2), and biochemical (secondary metabolites) analysis [30]. Optional parameters such as chromosome number may be recorded for new strains before deposition in the collection. This information is included in cell strain passports and maintained for future reference. Since 2021, the cultures in the collection have also been screened for antioxidant and antimicrobial activities. Strains with a specific growth rate >0.12 day^−1^ and composed of small-sized (about 50 µm) individual cells or small cell aggregates are preferable for bioreactor cultivation [33,60].

Newly developed cell strains often require optimization of medium composition, including phytohormones, inoculum density, and subculture duration to improve biomass and phytochemical yield. In addition, new strains usually undergo “auto-selection”—a process when highly-proliferating cells tend to survive and predominate in the population. Based on our experience, it usually takes one to two years for cell suspensions to stabilize under the optimized conditions, but this period is highly species-dependent. Stably growing strains with high content of the desired metabolites or high biological activities are further tested for bioreactor cultivation using a cascade of bioreactors (20 L–75 L–630 L) in the biotechnological facility of IPPRAS, where culture regimes (periodic or semi-continuous) and conditions (air supply, stirring rate) are further optimized. Large-scale (630-L) bioreactor production has been developed and routinely applied for suspension cell cultures of *Dioscorea deltoidea*, *Polyscias filicifolia*, *Panax japonicus*, and *Taxus wallichiana* [70,71,72,73]. Smaller 20-L or 75-L bioreactors were successfully tested for the cell cultures of *Tribulus terrestris*, *Taxus baccata*, *Polyscias fruticosa*, *Panax vietnamensis*, *Stephania glabra* (Roxb.) Miers, and some other species [66,74,75]. Some cell culture strains of biotechnological interest are presented in Table 1.

Several commercial products containing bioreactor-produced cell biomass are currently available in the market. For example, the food additive Vitagmal © is based on dried biomass of a *Polyscias filicifolia* cell culture which had passed the clinical trial and was approved for commercial use in the late 1990s. This phytopreparation was proven to exhibit adaptogenic and anti-teratogenic effects [76]. The cell culture of *Panax ginseng,* strain G1, was distributed to government companies for biotechnological production in the 1980s, but those pilot productions collapsed during the country’s economic crisis. In the 2020s, however, the strain was successfully adopted by a new commercial company currently producing a series of cosmetics and food additives on its base (https://cosmevita.ru/collections/, accessed on 24 January 2023).

**Table 1:** Some representative strains with valuable biotechnological traits from the core of the All-Russian Collection of Plant Cell Cultures.

Cell Strain	Year Strain Induced/Received by Collection	Characteristics
*Dioscorea deltoidea*, strains DM-05 and DM-05-03	1972/1985	Small-aggregated, rapidly growing cell strains developed through mutagenesis (single and double treatment with N-nitroso-N-methylurea) [77]; super-producer of steroidal glycosides protodioscin and deltoside and their 25(*S*)-isomers [60,78,79], adapted for large-scale bioreactor cultivation [80,81]. The total content of steroidal glycosides 4.6–5.7% DW [79] can be increased up to 13.9% DW in bioreactor production with a high aeration level [60,81]. Extensively used to study the regulation of steroidal glycoside biosynthesis in cell cultures [60]. Bioreactor-produced cell biomass was assessed for elemental composition [73], toxicology [82], and demonstrated positive effects in rats with induced type 2 diabetes mellitus and obesity [82,83].
*Polyscias filicifolia*, strains BFT-01-95 and Pf-SH	1991/1995; 2018/2023	Cell strains adapted for large-scale bioreactor cultivation [70] with a total content of polysciosides and their derivatives up to 3% DW. Bioreactor-produced cell biomass of BFT-01-95 has adaptogenic and anti-teratogenic activities, and is currently used in commercial food supplements [76,84].
*Panax ginseng*, strain G1	1959/1985	One of the oldest cell strains with stable growth and chromosome number, a producer of ginsenosides. The strain is currently used in the commercial production of several cosmetic products.
*Panax japonicus*, strain 62	1995–97/1998	Cell strain adapted for large-scale bioreactor cultivation [71] with a total content of ginsenosides (Rg_1_, malonyl-Rg_1_, Rb_1_, malonyl-Rb_1_, Rb_2_/Rb_3_, malonyl-Rb_2_/Rb_3_, Rd, malonyl-Rd, Rf, R_0_, chikusetsusaponin IVa) of 3.46% DW [85]. Bioreactor-produced cell biomass exhibited hypoglycemic and hypocholesterolemic activity in rats with diet-induced obesity [82].
*Tribulus terrestris*, strain 8	2014/2014	Cell strain adapted for laboratory bioreactor cultivation with a total content of furostanol glycosides 0.1% DW [75]. Bioreactor-produced cell biomass positively affected rats with induced type 2 diabetes mellitus and obesity [82,83].

Phytopreparations based on cell cultures of *Dioscorea deltoidea* (strain DM-05-03), *Panax japonicus* (strain 62), and *Tribulus terrestris* (strain 8) exhibited a range of positive effects in rats with induced type 2 diabetes mellitus and obesity [82,83]. The toxicological evaluation of *D. deltoidea* cell biomass [82] and its elemental composition [73] were the first steps toward certification for commercial application.

The collection is constantly acquiring new cell cultures. The priority is given to endemic and endangered medicinal species with proven use in traditional medicine. Most recent examples include the cell cultures of *Sutherlandia frutescens* and *Alhagi maurorum*, which are both medicinal plants of the Fabaceae family and contain both secondary metabolites and a unique composition of FAs [67,86], as well as cell cultures of *Ajuga turkestanica* and *Panax vietnamensis* with high antioxidant potential.

In conclusion, the All-Russian Collection of Plant Cell Cultures holds a valuable gene pool of cell culture strains of high biotechnological value and model strains for research and commercial application. This gene pool is a base for research and cell-culture biotechnology in-house and outside IPPRAS. The collection also provides services to research institutes and commercial companies by depositing cell strains for patent purposes, induction of new cell cultures based on the user’s interest, cell culture evaluation, passport development, etc.

## 3. Adventitious and Hairy Root Culture Collections

Adventitious roots (ARs) and hairy roots (HRs) cultured in vitro are other promising sources of pharmacologically important phytochemicals [87,88]. As organized tissues with high levels of differentiation, they hold an intermediate position between cell cultures and plants and, as such, are utilized as a model to study growth regulation and biosynthetic processes. Hairy roots are induced through the transformation of plant tissues with a Ri (root-inducing) plasmid from *Agrobacterium rhizogenes* Conn., a Gram-negative symbiotic bacterium that results in intensive branching of roots. Hairy roots can grow and proliferate on mediums without growth regulators, and are valued for stable metabolite production [87]. Adventitious roots are non-transgenic root cultures induced and maintained on a medium with auxins alone or in combination with low cytokinin concentrations [88,89]. Both HRs and ARs have been utilized as excellent sources of plant secondary metabolites, including ginsenosides, caffeic acid derivatives, anthraquinones, anthocyanins, hypericin, hyperin, eleutherosides, chlorogenic acid, tannins, and other compounds with high pharmacological value [90,91,92]. In addition, HRs can be used as factories for the production of recombinant proteins and for environmental restoration [93].

A collection of HR cultures was organized at IPPRAS in the 1980s [94] and currently includes 38 HR lines belonging to 25 plant species and nine families. The history, composition, and use of the HR collection have been recently reviewed [17].

**Figure 4:**
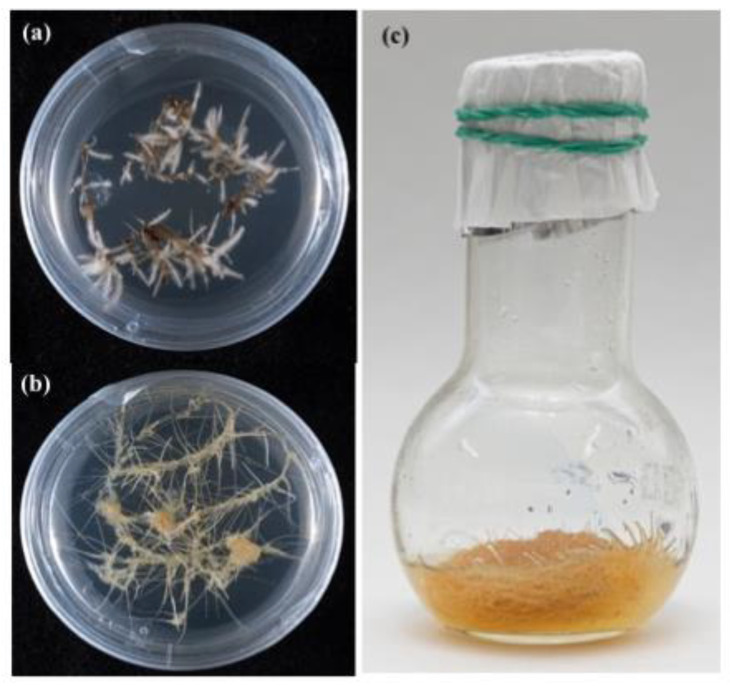
Adventitious root propagation: (**a**) *Digitalis lanata* Ehrh.; (**b**,**c**) *Maackia amurensis* on solid (**b**) and liquid (**c**) medium. (**a**,**b**) 25-day-old cultures; (**c**) 35-day-old culture. Photographs © IPPRAS.

The collection of ARs at IPPRAS was initiated in 2020 and is the “youngest” among the institute’s biotechnological collections. It includes 12 species of higher plants maintained on both agar-solidified and in liquid medium (Figure 4; Appendix A, Table A3). These cultures are currently being researched for the production of secondary metabolites and the biological activity of extracts.

The main advantage of HRs and ARs is their ability to produce secondary metabolites whose biosynthesis requires a certain level of tissue differentiation that does not exist in callus or suspension cell cultures [34]. For example, biosynthesis of alkaloids often involves different cell types and different compartments inside the cells, and the products can be translocated to other plant organs for accumulation [95]. As a result, the production of alkaloids in undifferentiated cell cultures may be difficult or unprofitable due to the low amount of the desired compounds. Therefore, the collections of isolated root cultures occupy a unique niche among the biotechnological collections in the IPPRAS bioresource conservation system. 

## 4. In Vitro Collection of Transgenic Potato Plants at IPPRAS

### 4.1. In Vitro Potato Collections in the World

Potato (*Solanum tuberosum* L.) is one of the most important food crops in the world. Still, its field cultivation is challenged by abiotic and biotic stresses (viruses, bacteria, fungi, insects), which dramatically reduce tuber yield [96,97]. Hence, many national and international institutions established in vitro collections of healthy potato materials, including breeding lines, modern cultivars, landraces, and heritage varieties. These aseptic plants are widely used in breeding and commercial production.

Steward and Caplin [98] first obtained actively proliferating tissue culture from the parenchyma of the potato tuber. In 1955, Chapman announced that he could regenerate the morphologically complete potato plant using the nodes excised from potato tuber sprouts [99]. Since then, a large variety of methods have been developed for the induction and maintenance of aseptic potato plant collections. Propagation methods using meristematic tissues, such as shoot tips [100], nodal cuttings [101], and microtubers are considered the most reliable for maintaining genetic identity during in vitro multiplication [102].

Many breeding and commercial seed-producing institutions maintain active and long-term (cryopreserved) collections of pathogen-free potato varieties. For example, the US Potato Genbank maintains 6000 accessions of 100 species of tuber-bearing relatives of *Solanum tuberosum* [103]. These accessions are also used for genetic and breeding experiments and commercial purposes [103]. The International Potato Centre (CIP, Peru) also maintains about 4388 accessions of cultivated and wild potato [104] that can be distributed to users worldwide. Additionally, the N. I. Vavilov All-Russian Institute of Plant Genetic Resources has one of the oldest and richest collections of wild and cultivated potato species, varieties, and interspecific hybrids with about 8500 accessions [105]. About 2932 potato accessions are safely maintained in aseptic plant collection at The Leibniz Institute of Plant Genetics and Crop Plant Research (Gatersleben, Germany) [24]. In addition, over 300 lines of both old and new cultivars of potato are secured in tissue culture and in cryopreservation conditions at the New Zealand Institute for Plant and Food Research [106]. A bank of healthy potato varieties was set up at the Russian Potato Research Center and provided many Russian regions with pathogen-free seed material [107].

The majority of the world’s potato collections prioritize the conservation of cultivated varieties and landraces, i.e., non-transgenic material. Unlike these collections, in vitro potato collection at IPPRAS focuses on transgenic lines that are primarily used in disease resistance research. This research has been initiated as part of the collaborative projects between IPPRAS, the Russian Potato Research Centre (Moscow, Russia), and the Institute of Genetics and Cytology at the National Academy of Sciences of Belarus (NASB) (Minsk, Belarus).

### 4.2. Collection of Transgenic Potato at IPPRAS

**Figure 5:**
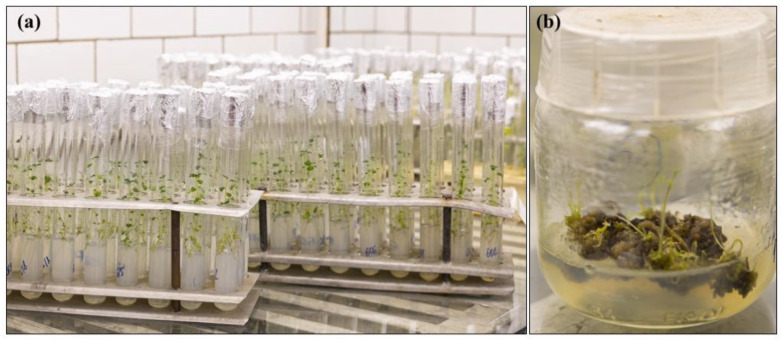
(**a**) In vitro collection of transgenic potato plants and original varieties; (**b**) regeneration of transgenic potato plants from leaf explants 86 days after inoculation.

The in vitro potato (*S. tuberosum*) collection of IPPRAS is relatively small but unique in terms of the conserved material: it is composed mainly by transgenic lines with a small proportion of the original commercial varieties (Table 2, Figure 5). This collection was primarily developed for research in potato transformation and production of disease-resistant potato varieties. The structure of the collection is presented in Table 2.

Forty-six original (non-transformed) commercial varieties of *S. tuberosum* were received from the Russian Potato Research Centre (Moscow, Russia [108]) and the Institute of Genetics and Cytology NASB. This initial collection was exploited to produce transgenic plants carrying reporter and disease resistance genes using transformation methodology developed at IPPRAS (patent RU 2 524 424 C1 [113]). Multi-year research resulted in five groups (subsets) of transgenic potato clones that have been further evaluated and, in several cases, returned to their respective institutions:

*Subset 2.1*, or the *GFP subset*, consists of nine transgenic lines of cv. Skoroplodny and Jucovsky Ranny expressing the GFP reporter gene. These lines were used to develop the express test for transgenic plant identification [109].

*Subset 2.2*, or the *SmAMP* subset, consists of four transgenic lines of cv. Udacha and eight transgenic lines of cv. Jucovsky Ranny. These clones express the Pro-SmAMP1 gene derived from *Stellaria media*, a weed plant that is used in human and animal diets in several countries [114]. All transgenic lines were annually screened for resistance to early blight (*Alternaria solani* and *A. alternata*) [110]. All the lines have been preserving the blight resistance trait for ten years and have been returned to the Russian Potato Research Centre for further research.

*Subset 2.3*, or the *NPT subset*, consists of nine transgenic lines of cv. Udacha and cv. Niculinsky expressing the NPT gene. These lines are primarily used as the controls in the studies.

*Subset 2.4*, or the *NsD subset*, contains transgenic lines of cv. Skoroplodny, cv. Jucovsky Ranny, and cv. Sarpo mira expressing the gene encoding defensin from black cumin, *Nigella sativa* [111]. This subset includes the following transgenic lines:

- Thirty-six transgenic lines of cv. Skoroplodny. Three lines demonstrated high blackleg resistance.
- Six transgenic lines of cv. Jucovsky Ranny. The original (non-transgenic) cultivar is susceptible to early blight. Out of the six transgenic lines, one line demonstrated high resistance to early blight during five years of annual infection tests.
- Forty-two transgenic lines of cv. Sarpo mira. Nine lines demonstrated high resistance to early blight and preserved this trait for five years, as confirmed by annual infection tests [112]. These perspective lines are currently used in the research programs at the Russian Potato Research Centre.

*Subset 2.5*, or the *NsD T1 subset*, includes T1 progenies produced by cross-pollination of NsD transgenic lines with potato varieties at the Institute of Genetics and Cytology NASB. In total, 81 T1 hybrids were screened for early blight resistance, and 42 clones with high resistance were returned to our collection.

These initial transgenic lines and their T1 progenies are now used at the Russian Potato Research Centre in the research programs and as initial materials for pathogen resistant potato varieties breeding. Since field cultivation of transgenic crops is currently not allowed in Russia, both transgenic potato lines and their T1 hybrids are grown and evaluated in special greenhouses. In the future, these lines may provide useful resistant traits for research and breeding programs and enhance our knowledge of early blight resistance mechanisms in potato.

## 5. IPPRAS Cryobank of Plant Genetic Resources

Cryopreservation, that is, the storage of viable cells and tissues in liquid nitrogen (LN, −196 °C) or LN vapor (−165 to −195 °C), is a convenient and efficient method for long-term conservation of plant genetic resources of both agricultural and endangered wild species as well as of biotechnologically important cultures [31,115,116,117,118]. The first cryo-collections of plant genetic resources appeared in global genebanks and research centers in the 1970s–1980s, and mostly functioned as a secure long-term backup for the materials maintained in active collections in vitro and in the fields [23,27]. Although introducing plant material to a cryobank is costly and laborious, it pays off over the years due to lower costs of maintenance and, most importantly, reduced chances for morphological, biosynthetic, and genetic variations in the specimens [119,120,121].

The first specimens of plant material in the cryobank at the Institute of Plant Physiology of the Russian Academy of Sciences (IPPRAS plant cryobank) were placed for long-term storage in LN in 1977 [122]. The success of the first experiments on freezing and thawing of the suspension cell cultures of wild carrot (*Daucus carota* L.) marked the start of the cryobank collections at our institute [123]. In 1982, Prof. Raisa G. Butenko, a corresponding member of the Russian Academy of Sciences, founded the new laboratory of plant cryopreservation at IPPRAS, which was led by Alexander S. Popov [124].

Investigation of freezing injuries and plant tolerance to low temperatures were traditionally the leading research directions at IPPRAS [125,126,127,128,129,130,131]. The decades of committed research resulted in the pioneering work by Nikolai Alexandrovich’s Maximov [125,126], who first discovered the cryoprotective properties of sugars, and is known as one of the founders of the ecological physiology of plants. These directions were further explored by a group of dedicated scientists, including Tamara I. Trunova, Oleg A. Krasavtsev, George A. Samygin, and Raisa G. Butenko, who continued to study cold response in plants under the leadership of Ivan I. Tumanov [127,128,129,130,131]. For the first time in Russia, this group initiated the research of plant material resistance to freezing in liquefied gases [122,128,131].

The application of in vitro culture technique was a significant step toward investigating and understanding the mechanisms of plant cell cryo-tolerance [129]. Importantly, in vitro cultures offered new ways of plant material dehydration and cryoprotection and broadened the range of specimens suitable for cryopreservation [122,123,132,133,134,135]. Since then, many publications repeatedly demonstrated that in vitro cultures opened up a way for establishing cryobanks of physiologically and genetically diverse plant materials [122,133,135,136,137,138,139].

Experimental data accumulated at the IPPRAS cryobank based on the research on cryo-tolerance of plants from different systematic groups eventually led to the development of new, broadly applicable cryopreservation protocols and customized programmable freezing equipment [122,123,140,141]. Some of these original innovations have been patented and used for cryopreservation of plant specimens belonging to diverse taxonomical groups and material types, including seeds, cell cultures, shoot meristems, protocorms, etc. (Table 3) [123,138,142,143,144,145,146,147,148,149,150,151,152,153,154,155,156,157,158,159,160].

IPPRAS cryobank collections are unique in terms of the diversity of specimens and represented taxa and the long-term preservation of viable plant material in LN. At the time of writing this review, many specimens have remained frozen for over 30 years. For over three decades, the cryobank has been enriched with numerous specimens representing rare and endangered plant species, valuable medicinal plants, and food crops. As a result, in 2018, the IPPRAS Cryobank of Plants (IPPRAS CBP) was given the governmental status of a unique scientific installation [124]. All specimens are stored in cryotanks of different volumes in LN. The choice of the cryopreservation technique depends on the type of plant material (Table 3).

**Figure 6:**
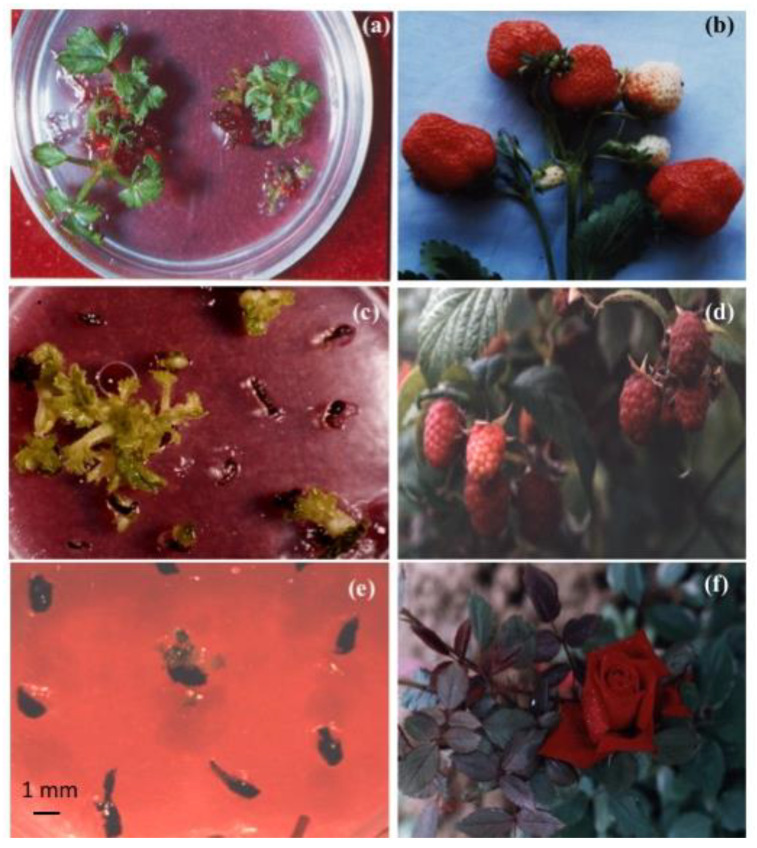
Recovery of various plant species after cryopreservation. Shoot apices were cryopreserved by slow-freezing using the programmed equipment of the IPPRAS cryobank. (**a**,**b**): *Fragaria x ananassa* Duch., cv. Kokinskaya pozdnaya (Russian strawberry cultivar, breeding by Alexander A. Vysotsky); (**c**,**d**): *Rubus idaeus* L., cv. Skromnitza (Russian red raspberry cultivar, breeding by Ivan V. Kasakov); (**e**,**f**): *Rosa* spp. (**a**) 1999, strawberry plantlets recovered from cryopreserved meristems (90% regeneration) with 40 days of in vitro culture (17 February 1999, protocol according to RF Patent № 2220563 [140,160]); (**b**) 2001, berries of strawberry plant regenerated from shoot apex cryopreserved in 1999; (**c**) 1999, raspberry shoots recovered from cryopreserved meristems (80% regeneration) with 40 days of in vitro culture (17 February 1999, protocol according to RF Patent № 2248121 [141]); (**d**) 2005, the fruiting of raspberry plants obtained from shoot apices after cryopreservation in 1999; (**e**) 2001, rose shoots recovered from meristems after slow-freezing cryopreservation (10% regeneration) (24 March 2001, protocol according to RF Patent № 2248121) with 30 days of in vitro culture; (**f**) 2004, the flowering of a rose plant developed from shoot apex after cryopreservation in 2001. Scans from pictures were obtained by film photo camera (Asahi Pentax Spotmatic F, Japan). © O.N. Vysotskaya.

**Figure 7:**
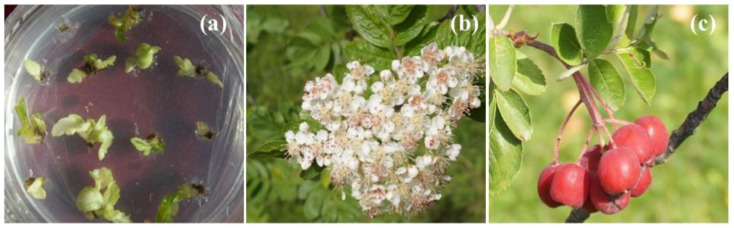
Recovery of rowan (*Sorbus* spp.) from meristems cryopreserved by fast-freezing technique after air dehydration (protocol according to Eurasian Patent № 036602 [153]): (**a**) 2006, shoots recovered from apical meristems after fast freezing in LN (21 December 2006); storage at −196 °C for 1 h; thawing and in vitro culture for 39 days (89% regeneration). (**b**) 2017, flowers on rowan tree developed from shoot apex cryopreserved in 2006. (**c**) 2021, fruits on rowan tree developed from shoot apex cryopreserved in 2006. (**a**) Scan from picture obtained by film photo camera (Asahi Pentax Spotmatic F, Japan); (**b**,**c**) pictures captured using Sony SLT A37. © O.N. Vysotskaya.

**Figure 8:**
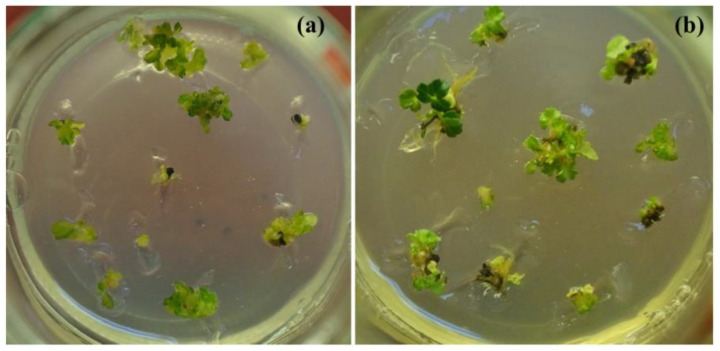
The comparison of strawberry culture regrowth after cryopreservation by the (**a**) classical slow-freezing method [160] and (**b**) the new fast-freezing technique developed at IPPRAS cryobank [153]: (**a**,**b**) apical meristems isolated from in vitro plantlets of strawberry (*Fragaria x ananassa* Duch., cv. Kokinskaya pozdnaya, in vitro mericlone from 1987). (**a**) 60% of meristems recovered growth after slow freezing by programmed equipment (patent RU 2 220 563 C1, [160]); 14 years of cryostorage (17 February 1999–19 September 2013) and cultured in vitro for 52 days. (**b**) 80% of meristems recovered growth after fast freezing in LN (protocol according to Eurasian Patent № 036602 [153]); 4 years of cryostorage (18 November 2009–19 September 2013) and cultured in vitro for 52 days. Pictures obtained by Sony SLT A37 on 11 November 2013. © O.N. Vysotskaya.

**Figure 9:**
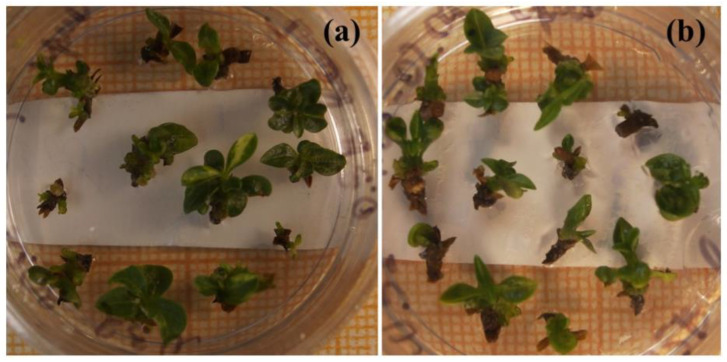
Recovery of lilac (*Syringa vulgaris* L.) plantlets from apical meristems after fast-freezing technique following air-flow dehydration (protocol according to Eurasian Patent № 036602, [153]) and two months storage at −196 °C (01 July 2022–30 August 2022): (**a**) cultivar Aucubaefolia; (**b**) cultivar Polina Osipenko. Shoot regrowth from 100% meristems during 60 days of in vitro culture. Pictures obtained by Sony SLT A37 on 07 October 2022. Photographer: O. V. Koroleva, Main Botanic Garden, Moscow, Russia.

**Figure 10:**
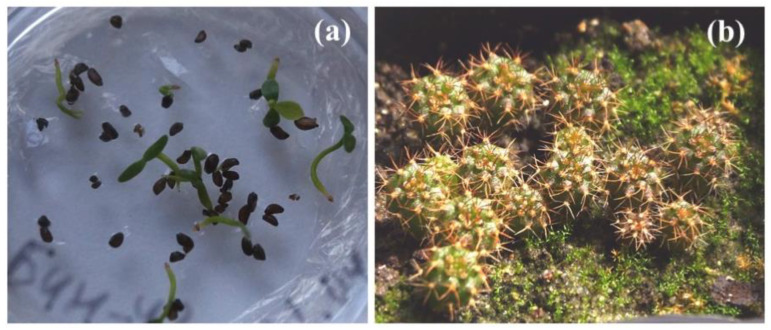
(**a**) 2016, 20% of bilberry (*Vaccinium myrtillus* L., Ericaceae) seeds germinated in vitro after storage in LN for over 24 years (1992–2016) [135]; (**b**) 2016, seedlings of *Frailea pulcherrima* (Arechav.) Speg. (Cactaceae) developed from 80% seeds germinated after direct freezing in LN and 183 days of LN storage (27 December 2015–19 January 2016). Seeds were germinated in a special soil mixture in the climate-control chamber [142]. Pictures were taken by Sony SLT A37 on 15 July 2016 (**a**) and 20 July 2016 (**b**). Photographer: A. Ju. Balekin, IPPRAS.

**Figure 11:**
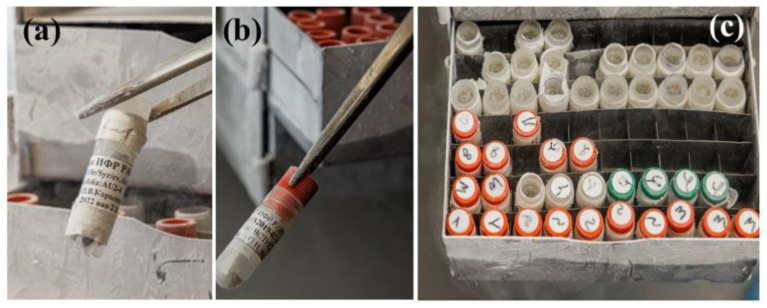
2022, IPPRAS cryobank specimens: (**a**) in front-cryotube with lilac shoot apices cryopreserved in 2022; (**b**) in front-cryotube with strawberry shoot apices cryopreserved in 2019; (**c**) cryotubes with different plant material in a cryo-rack. Photographs © IPPRAS, 16 February 2023.

The successful recovery of plant materials after years and even decades of cryogenic storage at IPPRAS cryobank and the retention of their main characteristics have been reported for in vitro clones and species, seeds, and cell cultures [123,134,135,143,152]. Some representative examples are presented in Figure 6, Figure 7, Figure 8, Figure 9, Figure 10 and Figure 11.

Healthy seedlings were obtained for various plant species after almost 30 years of cryopreservation [135]. The identity of the recovered plant materials to source specimens was assessed by comparing their agricultural and biotechnological traits and investigating them at the genetic level [149,150,154]. Our studies indicated no significant differences in the main characteristics between plant material of various types before and after cryopreservation [123,134,135,155,156] (Table 3). Plants regenerated from cryopreserved shoot tips were visually healthy without morphological abnormalities (Figure 6, Figure 7, Figure 8 and Figure 9) [145,150,154,155]. Strawberry plants regenerated from cryopreserved shoot tips were successfully acclimatized and produced fruits (Figure 6a,b) [140,152,155]. The stability of the recovered plant material was confirmed by using random amplified polymorphic DNA (RAPD), inter simple sequence repeats (ISSR), and retrotransposon-microsatellite amplified polymorphism (REMAP) analyses [149,150,154]. In addition, plants developed from cryopreserved shoot tips showed significantly higher multiplication rates compared to plants raised from the shoot tips that were not air-dehydrated and frozen in LN [150,154]. Cell cultures cryopreserved for periods varying from several months to decades completely restored their growth and biosynthetic abilities [134,157], including for cultivation in industrial bioreactors (Table 3) [70]. In addition, our research demonstrated that cryopreservation is useful not only for the long-term preservation of valuable plant material but also for selecting plant specimens with higher resistance to low temperatures [123,135,138,149,150,151,152,153,154].

Our experience gained through several decades of cryopreservation studies suggests that a high level of plant cryo-tolerance can be achieved by different cryopreservation methods and protocols once the optimum conditions for each material type are found [135,140,141,146,147,148,151]. For example, in addition to conventional methods, we have developed an effective cryopreservation technique for in vitro shoot meristems based on the fast freezing of specimens without using any toxic cryoprotectants or programmable equipment [135,151,153]. By utilizing this technique, we can dehydrate the shoot tips of strawberry and meristem tissues from in vitro plantlets of raspberry, blackberry, lilac, honeysuckle, and rowan tree under the flow of sterile air, frozen in LN, and recovered after several years of cryostorage (Figure 7, Figure 8 and Figure 9) [123,135,145,151,153].

Another broad research area explored at IPPRAS cryobank is seed cryopreservation of the endangered Russian plant species from Orchidaceae, Asparagaceae, Campanulaceae, Ericaceae, Liliaceae, Melanthiaceae, Caryophyllaceae, Poaceae and other families (Figure 10) [135,143,148,158,159]. This work is performed in close collaboration with the Main botanic garden (Moscow, Russia) and the Apothecaries garden at Moscow State University. 

In conclusion, researchers at IPPRAS cryobank have developed an effective system for long-term preservation of the collections of vegetatively propagated plant material that integrated the method of in vitro culture, cryopreservation, and ex vitro cultivation [135,152,153,155]. Using this system, the cryobank can store strains for the long term and effectively use numerous clones of ornamental, berry, and fruit plants for several decades [123,134,135,145]. Between 1977 and 2022, the IPPRAS cryobank was enriched with more than 1000 specimens (cell cultures, shoot meristems, and seeds) of different plant material belonging to 457 species of higher plants from 74 families [122,123,134,135,153].

Our developments have been well tested in practice but can also be used in fundamental scientific research (the study of genetic and morphological diversity of plants), for preservation and propagation of pathogen-free plant material in agriculture, production of medical substances, and in education as well as for the conservation of the endangered species and reintroduction into their habitats. It is important that, unlike many other world cryobank facilities, the IPPRAS cryo-collection not only serves to safely back up the institute’s existing active collections but also acts as an independent research unit. The cryobank operates its research programs and has developed long-term connections with external users and collaborators among botanic gardens, agricultural research institutes, and universities. These academic collaborations, and a long history of dedicated research work, resulted in a large diversity of cryopreserved specimens and unique results in monitoring the stability of plant materials following long-term cryopreservation.

## 6. Culture Collection of Microalgae and Cyanobacteria IPPAS at the Institute of Plant Physiology of Russian Academy of Sciences

The first collections of algae maintained under laboratory conditions were established in the late-19th–early 20th centuries, driven by a growing scientific interest in algae in general and the need for unlimited access to specific strains [161]. Pioneer studies on algae cultivation by initial phycologists, including M. W. Beijerinck (1851–1931), R. Hodat (1865–1934), E. G. Pringsheim (1881–1970), V. Uhlíř (1892–1915), and S. Prát (1895–1990) laid the foundation for algae cultivation methods and establishing the algal collections; some of these first collections are still existing nowadays [161]. Microalgae culture collections established worldwide possess high ecological, environmental, taxonomic, and biotechnological value. The oldest, largest, and most internationally requested collections of microalgae, to name a few, are Culture Collection of Algae and Protozoa (CCAP) in the UK (>2100 strains, [162]), Culture Collection of Algae at Göttingen University (SAG) in Germany (about 2300 strains, [163]), Pasteur Culture Collection of Cyanobacteria (PCC) in France (>750 strains, [164]), Culture Collection of Autotrophic Organisms (CCALA) in Czech Republic (>1000 strains, [165]), and Culture Collection of Algae of the University of Texas (UTEX) in USA (>3000 strains, [166]).

Of the 27 Russian algae culture collections, the “Culture collection of microalgae and cyanobacteria IPPAS” is one of the oldest and the most diverse collection of biotechnologically important and model strains. The collection is open for collaborations, and aims to provide targeted services and assistance to scientists working on the physiology and biochemistry of photosynthetic microorganisms or biotechnological applications of microalgae and cyanobacteria.

### 6.1. Culture Collection of Microalgae and Cyanobacteria IPPAS—Historical Perspective and Composition

The culture collection of microalgae and cyanobacteria was established at the Institute of Plant Physiology of USSR Academy of Sciences (now known as Russian Academy of Sciences) by Dr. Maya G. Vladimirova in 1958 under the supervision of Prof. Victor E. Semenenko. The research of Prof. Semenenko and his group was primarily focused on the application of the microalgae in closed biological life support systems, i.e., in space programs that were actively developing at their time. Due to the broad research interests of Prof. Semenenko and his team, the research area was expanded largely beyond space biology. The new research directions initiated by his group covered principles of self-regulation and high plasticity of microalgal metabolism, mechanisms of photosynthesis regulation, and the mechanisms of concentration, generation, and fixation of carbon dioxide (CO_2_) in the chloroplasts of algae and in cyanobacteria. These studies of Prof. Semenenko laid the solid platform for microalgae biotechnology in Russia (see [167] and references herein).

The research interests of the founders determined the main purpose of the collection and consisted of the following areas: the preservation and development of the gene pool of highly productive microalgal strains, the selection of strains producing valuable compounds for biotechnological application, and fundamental research in photosynthetic cell biology [168]. Nowadays, the main activities of the collection are maintaining and increasing the gene pool of non-pathogenic natural and genetically modified strains of cyanobacteria and microalgae—potential producers of valuable metabolites and models for fundamental research; providing strains to scientific, educational, and biotechnological organizations; taxonomic identification of collection strains; optimizing storage and growth conditions; developing methods for purification of incoming strains; and evaluating their biotechnological potential.

**Figure 12:**
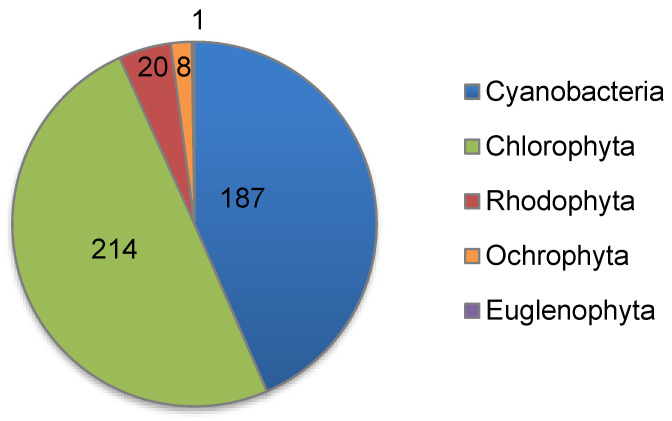
Taxonomic diversity of the collection of microalgae and cyanobacteria IPPAS.

**Figure 13:**
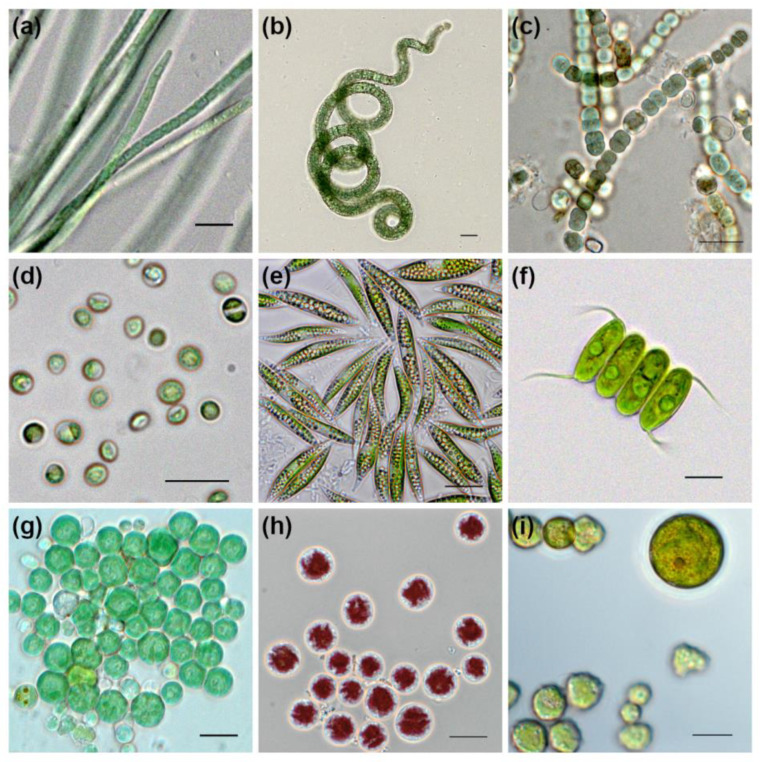
Morphological diversity of microalgae and cyanobacteria strains maintained at the collection. Cyanobacteria strains (**a**–**c**): (**a**) *Desertifilum tharense* Dadheech and Krienitz IPPAS B-1220; (**b**) *Limnospira* sp. Nowicka-Krawczyk, Mühlsteinová and Hauer IPPAS B-1526; (**c**) *Dolichospermum* sp. (Ralfs ex Bornet and Flahault) P. Wacklin, L. Hoffmann and J. Komárek IPPAS B-1213. Green algae strains (**d**–**f**): (**d**) *Chlorella sorokiniana* Shihira and R. W. Krauss IPPAS C-1; (**e**) *Ankistrodesmus falcatus* (Corda) Ralfs IPPAS A-217; (**f**) *Desmodesmus communis* (E. Hegewald) E.Hegewald IPPAS S-313. Red algae strains (**g**,**h**): (**g**) *Cyanidium caldarium* (Tilden) Geitler IPPAS P-510; (**h**) *Porphyridium cruentum* (S. F. Gray) Nägeli IPPAS P-273. Ochrophyta strain (**i**) *Vischeria punctata* Vischer IPPAS H-242. Scale bars are 10 µm.

As of 2022, the collection holds 430 strains, including 243 strains of eukaryotic microalgae and 187 strains of cyanobacteria belonging to 91 genera and 106 species (Table 4, Figure 12 and Figure 13). Eukaryotic algae are represented by phyla Chlorophyta, Rhodophyta, Ochrophyta, and Euglenophyta. Since 1989, the collection has been a member of the European Culture Collections’ Organisation (ECCO) [169], and it is registered with the World Data Centre for Microorganisms (WDCM) under number 596 [170] with the acronym IPPAS (an acronym for the Institute of Plant Physiology of Academy of Sciences USSR), which is still in use today despite the change of the host institute’s name to IPPRAS in 1991. The same acronym (IPPAS) is continuously used in the codes of most strains in the collection.

The strains in this collection originate from 33 countries and from all continents except Antarctica; most strains are from Russia and USA, followed by Vietnam, Mongolia, Kazakhstan, Switzerland, the Czech Republic, and other countries (Appendix A, Table A4). The basis of the collection was formed in 1957–1964 when the first strains were purposefully obtained, mainly from the algal collection of Charles University in Prague and a working collection of Dr. Hiroshi Nakamura from the Microalgae Research Institute of Japan. Thirty-one of those original strains are still maintained in the collection. Until the 2010s, the new strains had been mostly acquired from other governmental collections and working collections of individual scientists [168]. Since 2014, the number of strains collected and isolated by the collection’s staff has constantly increased, reaching 56 strains in 2022 (Appendix A, Table A5).

Microalgae and cyanobacteria strains should meet specific criteria to be deposited in the collection [172]. Most of these criteria relate closely to the biotechnological focus of the collection. These include high photosynthetic activity when growing on mineral-rich media, effective growth under high light intensity, thermophilic or thermotolerant physiology, and resistance to contamination by microorganisms (bacteria, molds, and other algae). Unicellular strains and strains with small colonies are preferred for intensive large-scale cultivation. However, the undesirable traits include the culture’s ability to produce slime, the tendency to aggregate, and adhesion to surfaces. The culture’s life cycle should be simple, without sexual reproduction or motile stages. Strains with vegetative reproduction are usually more genetically stable.

Strains that match these criteria can be obtained from the collected samples using the culture enrichment method. For the enrichment cultivation, nutrient-rich media, such as BBM-3N [173], BG-11 [174], Tamiya [175], and Zarrouk’s media [176], are used. Cultures are grown under continuous illumination of 30–50 μmol photons m^−2^ s^−1^ at 22, 27, or 32 °C. Under these conditions, the cultures become enriched with fast-growing mesophilic or thermophilic strains which do not require light/dark change for proliferation. Individual strains are isolated from the enriched culture by serial dilutions and colony picking or by direct cell isolation using an inverted microscope [177].

Many strains in the collection are unique extremophiles: thermophiles from hot springs, psychrophiles—symbionts of Baikal and White Sea sponges, and inhabitants of snowdrops and cold waters, halophiles from saline lakes, acidothermophiles from volcano calderas, and haloalkaliphiles from soda lakes. These organisms represent a valuable yet underexplored resource for fundamental science (studying stress adaptation mechanisms) and biotechnology (as producers of unique metabolites).

The majority of the collection strains are wild forms. There are also ~90 mutants of cyanobacterial model organisms *Synechocystis* sp. Sauvageau and *Synechococcus elongatus* (Nägeli) Nägeli with regulatory and metabolic gene knockouts and ~50 photosynthetic mutants of model green algae *Chlamydomonas reinhardtii* P. A. Dangeard. The strains available for distribution are presented in the online catalog [178].

**Figure 14:**
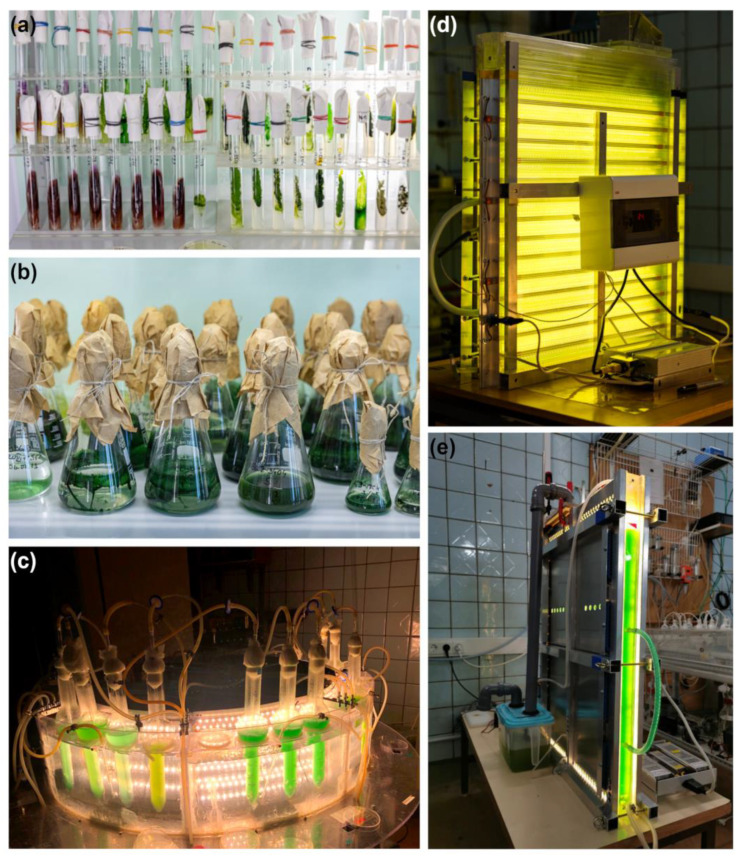
Maintenance and cultivation of microalgae and cyanobacteria strains. (**a**) slants; (**b**) flasks; (**c**) laboratory system for intensive cultivation; (**d**,**e**) flat panel photobioreactors, FP-17 and FP-18, respectively. Photographs (**a**–**c**)—© IPPRAS; (**d**,**e**)— © David Gabrielyan, IPPRAS.

Most strains are maintained by periodic subculturing, which is the cheapest but laborious method. Both liquid and agar media (slants and Petri dishes) are used (Figure 14a,b). Based on our experience, liquid media is preferable for strains with specific requirements for low (2–4) or high (9–11) pH or high salt concentration. For most of the strains growing on agar media, the subculturing cycle is divided into two parts: at first, after transfer to a fresh media, the cultures are grown for 2–4 weeks under continuous illumination of 30–50 μmol photons m^−2^ s^−1^ at temperatures close to optimal (22–27 °C for mesophiles and 32 °C for thermophiles); then, the cultures are stored until the next transfer at low light (10–20 μmol photons m^−2^ s^−1^) and lower temperatures (10–12 °C for mesophiles and 22 °C for thermophiles). The storage at low temperatures allows increasing each subculturing cycle up to 7 months compared to 2–3 months for cultures stored at room temperature.

### 6.2. Use of the Collection Strains for Fundamental Studies on the Physiology of Photosynthetic Microorganisms

Alongside strains of high biotechnological value, our collection maintains popular model strains used mainly in fundamental studies. For example, strains of *Chlamydomonas reinhardtii* (IPPAS D-221 and IPPAS L-1021) [179], *Sodalinema gerasimenkoae* Samylina, Sinetova, Kupriyanova and Tourova (former *Microcoleus* sp. Desmazières ex Gomont) IPPAS B-353 [180], and *Crocosphaera subtropica* Mareš and J. R. Johansen (former *Cyanothece* sp. (Nägeli) Komárek) IPPAS B-1603 [181] have been used in the past decade for intensive studies of the CO_2_-concentrating mechanism (Table 5). Mechanisms of intracellular regulation and stress responses in photosynthetic cells have been investigated using wild-type strains, and about 90 regulatory mutants of *Synechocystis* sp. PCC 6803 are now deposited in the collection [182,183,184,185,186]. These studies revealed that cyanobacteria operate a variety of systems that perceive a signal and regulate gene expression in response to various stresses (low and high temperatures, salt, hyperosmotic, light, and oxidative stresses). These include two-component regulatory systems, eukaryotic-type serine–threonine protein kinases, the σ-subunits of RNA-polymerase, DNA-binding transcription factors, and DNA supercoiling [182,183]. Furthermore, a systemic analysis of *Synechocystis* sp. PCC 6803 transcriptomes under stress conditions revealed two distinguished groups of stress-induced genes: one related to heat-shock proteins (HSPs), which are activated by all stresses except cold, and the second group comprised genes induced by all stress factors except heat shock. The analysis demonstrated that ROS accumulation and redox state changes are universal triggers for stress responses in cyanobacteria [184,185,186].

### 6.3. Biotechnological Application of Collection Strains and Links to Biotechnological Production Facilities

The biotechnological potential of algae and cyanobacteria strains is being evaluated by assessing their growth characteristics, biochemical composition, and genome analysis.

Growth is characterized by maximal specific growth rate and productivity. Since the strains in the collection are mainly intended for biotechnological use, it is important to optimize chemical and physical conditions to achieve their maximal growth and productivity. The main parameters include temperature, light intensity, CO_2_-concentration in the gas mixture, and medium composition. For this purpose, the Laboratory system for intensive cultivation (LSIC) was designed [187] (Figure 14c). LSIC is a small-scale equipment simulating culture conditions that are very close to large-scale cultivation, and allows parallel testing of different conditions with minimal costs. This system thoroughly controls the temperature, light, and CO_2_ contents. Additionally, LSIC can simultaneously run an experiment with eight different conditions varying in light, temperature, and CO_2_-concentration in four replications for each condition set. LSIC has been used for screening strains suitable for mass cultivation, for growth optimization, and for studying the biochemical composition of microalgae cells at different stages of growth and selection of the most productive strains [187].

Some strains with robust growth characteristics, such as *Chlorella sorokiniana* IPPAS C-1 and *C. vulgaris* Beijerinck IPPAS C-2 have been used as reference strains for different biotechnological applications [175,196,197,198,199,200] (Table 5), including the design and testing of the novel models of photobioreactors [175,198] (Figure 14d,e). *Synechocystis* sp. IPPAS B-1400 (PCC 6803 GT-L) was used for the development of a new method for growth characterization and optimization [210] and for studying quantitative growth properties and resource allocation [211].

As a part of the biochemical screening, biochemical composition (protein, lipid, and carbohydrate content; FA composition of total lipids; chlorophyll and carotenoid content) has been evaluated for a diverse range of strains at different growth stages. Using this approach, we selected strains with high potential for the production of biofuels, pigments, feed, food additives, and ω-3 polyunsaturated FAs, including eicosapentaenoic acid (EPA) [176,202,212] (Table 5). FA analysis revealed cyanobacterial strains with unusual FAs, such as myristic (14:0) and myristoleic acids (14:1Δ9) [176] or 7,10-hexadecadienoic (16:2Δ7, 10) acid [207] (Table 5).

The draft genomes of five cyanobacterial strains from the collection were sequenced and analyzed [205,208,213,214]. Information on the FA composition and the set of desaturases revealed from genomic data was further used to develop the biochemical classification system for cyanobacteria [215]. The combination of genomic and biochemical data led to a discovery of a new desaturase with unique properties [206] and the understanding of the mechanisms of double bond formation by the known desaturases [216]. This knowledge of cyanobacterial desaturases and their mode of action may be extended to desaturases expressed in edible plants and, eventually, may be useful to improve plant cold stress tolerance [217].

### 6.4. Taxonomic Identification

Taxonomic identification is necessary for understanding and predicting the biotechnological potential of the strains. Taxonomical studies are also helpful for systematic revisions of the collection strains and clarifications of their origin. Genetic markers, such as small ribosomal RNA sequences and ITS region sequences, are used for phylogenetic tree constructions. In this collection, we have developed and applied an integrative approach for strain identification which combines genetic data with additional data on morphology, ultrastructure, physiology, and biochemistry. Using this approach, new strains of *Cyanobacterium* sp. IPPAS B-1200 [176] and *Desertifilum tharense* IPPAS B-1220 were described for the first time based on their place of origin [207,218]. Furthermore, three new species of the genus *Sodalinema* Cellamare, Duval, Touibi, Djediat and C.Bernard were described: *S. orleanskyi* Samylina, Sinetova, Kupriyanova and Tourova (IPPAS B-2037), *S. gerasimenkoae* (IPPAS B-353), and *S. stalii* Samylina, Sinetova, Kupriyanova and Tourova (IPPAS B-2050). Additionally, their taxonomic position in the “marine *Geitlerinema*” group was clarified [209]. Among green algae, a new species (*Micractinium thermotolerans* Krivina et al.) and a new genus and species (*Neochlorella semenenkoi*) were described [203,219].

In conclusion, the available and constantly growing holdings of the collection of microalgae and cyanobacteria IPPAS enable the preservation of gene pools of species and strains from different habitats and climatic zones. This collection is a platform for research, educational, and public awareness activities and provides the basis for innovative biotechnological projects. It can supply research material with specified properties and guarantees the preservation of new microalgae strains with high biotechnological potential obtained from project work in other organizations. Overall, the collection is an important resource for fundamental and applied research, especially in biotechnology, which is actively developing in Russia and worldwide.

## 7. Quality Management System (QMS) at IPPRAS Collections

### 7.1. Documentation and Information Management

All in vitro culture collections at IPPRAS follow strict information collection and storage procedures. Every collection maintains its database, including passport data for each strain, subculture, and distribution records. The passport data follow the international system modified for the collection needs and contains, *at minimum*, information about the strain origin and morphological, physiological, biochemical, and molecular identification characteristics (for algae and cyanobacteria). The typical passport for plant cell cultures, for example, contains information on cell morphology, aggregation type and sizes, medium composition, optimum culture conditions (inoculum density, subculture period, etc.), growth curves, chromosome number, data on secondary metabolite production (for producer strains), information on cryopreservation, and any additional observations. All passports are regularly updated; specifically, the growth characteristics of the cell cultures are re-assessed every five to ten years.

All operations in the collections are performed according to standard operation procedures, regularly updated, and recognized by all staff. These include the procedures of medium preparation, subculture, contamination monitoring, strain identification, etc.

### 7.2. Contamination and Purity Control

Maintenance of in vitro culture collections implies regular manual transfers to a fresh medium, which may potentially lead to contamination or mislabeling [220]. Therefore, regular checks for contamination and purity are essential for collection maintenance.

In vitro collections of cell and root cultures are monitored once a week for potential contamination by fungi or bacteria, which instantly become visible due to rapid growth on a sucrose-containing medium. The contamination rate in these collections is close to zero, e.g., during the past five years, only three individual flasks were contaminated by fungi and this contamination did not affect the maintenance of cell lines due to regular duplications. Purity tests in the collection of microalgae and cyanobacteria are performed under a microscope and by using agar test media, which is identical to the usual maintenance media but does also contain 0.2% of glucose and 0.02% of casamino acid. The test plates are incubated for 2–4 weeks in darkness at room temperature so the contaminant growth may become apparent. Only some strains are axenic; hence, moderate bacterial contamination is acceptable, but this information must be indicated in the strain card. Fungal contamination is unacceptable and, once detected, is treated with carbendazim as recommended [221].

In addition, the culture collection of microalgae and cyanobacteria involved regular strain authenticity and purity testing to avoid contamination by other microalgae or cyanobacteria and mislabeling [220]. The data collected are compared with reference information collected and maintained for each strain, including photographs for cell morphology and the sequences of DNA markers: the 16S rRNA gene and ITS region for cyanobacteria, and the 18S rRNA gene and ITS1 and ITS2 regions for eukaryotes. The collection and storage of the reference database are essential tasks. The microscopy tests to confirm the strain authenticity are performed once every two years or when the strain is prepared for distribution.

Modern genetic tests helped to reassess the origin and authenticity of some old strains in the collection and remove duplications. For example, in recent years, genetic tests confirmed that the *Vischeria punctata* IPPAS H-242 strain acquired in 1958 was genetically identical to the strain SAG 887/1 derived from the same original strain of Vischer [212]. In addition, the strains *Chromochloris zofingiensis* IPPAS C-30 and IPPAS C-108 were confirmed to be genetically identical to the strain SAG 211-14; all three strains originated from the strain of *C. zofingiensis* Dönz, 205, 1933.

In plant cell culture collection, strain identification using genomic methods is difficult due to the larger genome size in higher plants. Recently, a pilot study using sequencing methods was initiated to check the presence of potential endophytes in the collection, but this study is in the early phase.

### 7.3. Collection Duplication and Safety Back-Up at −70 °C and −196 °C

The duplication system is an essential part of the collections’ QMS. The core strains of plant cell culture collections are duplicated as both callus and suspension cell cultures. In addition, the core collection of the most biotechnologically important producer strains is independently maintained at the bioreactor production facility. All microalgae and cyanobacteria strains on agar slants are preserved in at least two replicates of the new subculture and two replicates of the previous one.

It is acknowledged that, although diverse types of plant samples and algal isolates have been successfully maintained by subculturing for decades, in vitro maintenance in artificial conditions may pose selective pressure and cause a genetic drift [222]. Some phenotypic characteristics of microalgal and cyanobacterial strains, such as spine production, optimum growth rate, ketocarotenoid and phycobiliprotein content, alkaloid neurotoxin production, and gas vacuole presence, were reported to be unstable during serial subculturing [223]. On the other hand, several studies, including ours, could not detect any genomic differences between duplicated strains of the same isolates maintained by continuous subculturing under different conditions and even in different collections over decades [187,212,224,225].

A possible solution to reduce the risks caused by continuous subculturing mentioned above is to maximize the interval between subcultures or apply low-temperature storage. Hence, optimizing storage conditions to maintain strain viability for longer periods with less frequent subcultivations remains one of the top research areas in the collections.

The best solution for maintaining the genetic stability of strains is cryopreservation, i.e., the storage at temperatures below −130 °C, when chemical activities cease and ice recrystallization is prevented [222]. Several producer plant cell strains were successfully cryopreserved in the 1980s–1990s [123,133,226]. In the time period between 2010 and 2020, several cell strains were recovered after being cryopreserved for 5–27 years. Survival above 20% was sufficient to re-establish cell suspensions. Cell culture of *Medicago sativa* fully restored its growth, mitotic index, and peroxidase production after 27 years of cryopreservation [134]. The growth parameters of *Polyscias filicifolia* cell suspension recovered after five years of cryopreservation and cultured in 20-L, 75-L, and 630-L bioreactors were slightly higher compared to those of the same cell strain maintained by periodic subcultures for the same period [70]. Two *Rhaponticum carthamoides* cell culture strains completely restored their growth indices and protoberberine content after several months in LN [157]. Likewise, the diosgenin, sitosterol, and stigmasterol content in *Dioscorea deltoidea* cell cultures remained unchanged after cryopreservation [133].

The algae and cyanobacteria collection currently utilizes mid-term preservation at –70 °C to back up some strains from the active collection. *Synechocystis* mutant strains, model cyanobacteria *C. subtropica* IPPAS B-1603, *Acaryochloris marina* IPPAS B-1601, and type strains of novel cyanobacterial and green algal species [203,209,219,227,228,229,230] are preserved at −70 °C in a freezer with 10% dimethyl sulfoxide or 10% glycerol as cryoprotectors, as described by Iwamoto et al. [231]. The strains *C. subtropica* IPPAS B-1603 and *Spongiosarcinopsis terrestris* IPPAS C-2041 were successfully recovered after several months of such storage, and *A. marina* IPPAS B-1601 was recovered after five years.

## 8. User Services

The IPPRAS collections offer their users a diversity of in-kind and paid services (Appendix A, Table A1), including, but not limited to the following services:

- live material distribution;
- deposition and secure storage of strains developed by users;
- induction/isolation of new strains for specific applications;
- culture evaluation, optimization, and passport development.

Living algal and plant cell cultures are mainly distributed for research purposes as well as for biotechnological and educational applications. During the past decade, the microalgae and cyanobacteria collections received 10–15 orders and distributed up to 100 strains annually. For cell culture collection, these numbers are close to 5–6 strains per year. In addition, the cell and algae collections of IPPRAS deposits the biotechnologically relevant strains for patent purposes. Most of the collections’ users are affiliated with Russian research and educational institutions, including the host institute, and Russian biotechnological commercial organizations. The algae and cyanobacteria collection also distributes the strains internationally.

The potato plant collection closely collaborates with the Russian Potato Research Centre (Moscow, Russia) and the Institute of Genetics and Cytology at the National Academy of Sciences of Belarus (Minsk, Belarus) with regular material exchange. The IPPRAS cryobank, in addition to multiple long-term research collaborations, serves as an ultimate backup for several collections of ornamental, agricultural, and endangered plants developed at Russian research institutes and botanic gardens.

## 9. Conclusions

**Figure 15:**
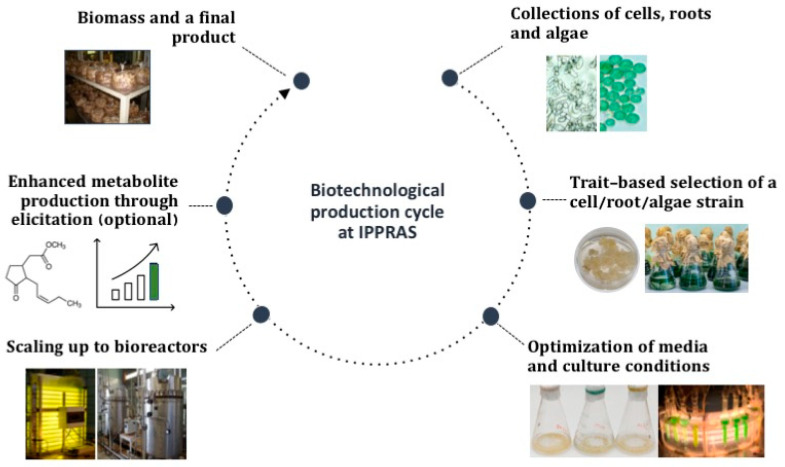
The linkage of plant cells, algae, and cyanobacteria culture collections with biotechnological facilities at IPPRAS. The first three steps of the biotechnological production cycle are accomplished by the collections.

In vitro and cryopreserved collections of plant material, algae, and cyanobacteria hosted at IPPRAS are unique regarding both diversity of the preserved taxa and the duration of storage. Most collections were created in the 1950s–1970s, and some of those original strains are still maintained. Large part of the collections’ funds are available for research and distribution. Unlike many other in vitro and cryobank collections serving mainly for safety backup storage, IPPRAS collections are independent research units running multiple scientific programs with collaborators inside and outside the host institute. Each collection holds unique genetic resources that are extensively utilized in both fundamental and applied research, for biotechnological production of biomass rich in nutrients and/or medicinally important metabolites, conservation of endangered species, education, raising public awareness, and other purposes. The total collection holdings sum up to 430 strains of algae and cyanobacteria, over 200 lines of in vitro potato clones, and over 150 strains of cell cultures, hairy and adventitious root cultures. These collections, including the cryobank, are not museums maintaining their holdings in perpetuity. They are dynamic, evolving systems that constantly exchange and acquire new material; develop and evaluate new strains; and perform their taxonomical, nutritional, and pharmacological screening; design and optimize new conservation methods. The knowledge and competencies acquired through decades of conservation resulted in the development of novel methodologies, such as the integrative approach for algae strain evaluation and a new cryoprotectant-free cryopreservation method for plant materials. Close cooperation between germplasm collections and bioreactor facilities makes IPPRAS the only institute in Russia operating a self-sustaining biotechnological production cycle (Figure 15) from the new strain development to a final product—cell or algal biomass with standardized characteristics. Together, the collections form a bioresource and information hub that connects IPPRAS with partner institutions and attracts students, funds, and research cooperation. Recently, the collections started to implement genotyping, transcriptomic, and metabolomic analysis for strain evaluation. In addition, we are working on developing novel algae- and plant cell-based commercial products, attracting new commercial and research partners, and participating in public awareness events.

## 10. Patents

Jur’eva N.O.; Egorov T.A.; Beljaev D.V.; Sobolkova G.I.; Derevjagina M.K.; Rogozhin E.A.; Tereshonok D.V.; Meleshin A.A.; Shelukhin P.G. Method of production in vitro of potato forms resistant to phytophthora and Alternaria spot causative agents. RU 2 524 424 C1. 27.07.2014 Bull. 21. Federal’noe gosudarstvennoe bjudzhenoe uchrezhdenie nauki Institute fiziologii rastenij im. K.A. Timirjazeva Rossijskoj Akademii nauk.

Vysotskaya, O.N. The technique for cryopreservation of meristematic shoot tips isolated from plants in vitro. Eurasian Patent no. 036602 (RU). Prior.: 13 July 2019.: 27 November 2020. In Russ.

## Figures and Tables

**Table 2:** Collection of in vitro potato plants at IPPRAS.

Subset	Cultivars	Provider of the Original Material	No. of Lines	Foreigner Genes Expressed	Use	Reference
Non-transformed						
1	Cultivated Russian varieties, haploid inducer line, dihaploids and foreign commercial cultivars	Russian Potato Research Centre (Russia), Institute of Genetics and Cytology NASB (Belarus)	46	No	Initial material for transgenic plant production	[108]
Transformed						
2.1.	cv. Skoroplodny, cv. Jucovsky Ranny	Russian Potato Research Centre	9	hGFP reporter gene	Express-test for transgenic plant identification	[109]
2.2.	cv. Udacha, cv. Jucovsky Ranny	Russian Potato Research Institute	12	Pro-SmAMP1 gene	Breeding programs: early blight resistance	[110]
2.3.	cv. Udacha, cv. Niculinsky	Russian Potato Research Centre	9	NPT gene	Controls in experimental studies	no
2.4.	cv. Skoroplodny	Russian Potato Research Centre	36	NsD3 (from *Nigella sativa* L.), a defensin-encoding gene	Blackleg resistance studies	[111]
	cv. Jucovsky Ranny (early blight susceptible)	Russian Potato Research Centre	6	NsD3 (from *Nigella sativa*), a defensin-encoding gene	Early blight resistance studies	[112]
	cv. Sarpo mira	Russian Potato Research Centre	42	NsD3 (from *Nigella sativa*), a defensin-encoding gene	Early blight resistance studies	[112]
2.5.	T1 hybrids	Institute of Genetics and Cytology NASB	42	NsD3 (from *Nigella sativa*), a defensin-encoding gene	Breeding programs: early blight resistance	no

**Table 3:** Recovery of different plant materials after storage at IPPRAS cryobank.

Taxon	Material Type	Method of Cryopreservation	Period of Storage	Viability/Regrowth after Cryopreservation	Traits Evaluated	Reference
*Medicago sativa*	Cell culture	PF	27 years	20%	The growth index, mitotic index, and peroxidase activity were fully restored	[134]
*Polyscias filicifolia*	Cell culture	PF	5 years	40%	Growth parameters in 20-L, 75-L, and 630-L bioreactors were fully restored	[70]
*Rhaponticum carthamoides* and *Thalictrum minus*, two strains	Cell culture	PF	Several months	60–80%	The growth index and protoberberine content were fully restored	[157]
*Dioscorea deltoidea*	Cell culture	PF	Several months	30%	The growth index and production of diosgenin, sitosterol, and stigmasterol were fully recovered	[133]
Orchidaceae, 130 species	Seeds	Fast-freezing	Several days to several years	0–100%	In vitro germination	[135,144,146,147,148,158,159]
Asparagaceae, Campanulaceae, Ericaceae, Iridaceae, Fabaceae, Liliaceae, Melanthiaceae, Poaceae	Seeds	Fast-freezing and PF	Several days to 30 years	0–100%	In vitro and in soil germination	[135,143]
Cactaceae	Seeds	Fast-freezing	22 days	23–81%	In soil germination	[142]
*Fragaria*, 28 cultivars	In vitro shoot meristems	PF	1 h to 36 months	0–100%	In vitro recovery	[140]
*Fragaria*, 50 cultivars	In vitro shoot meristems	Fast-freezing	5 to 10 years	75–100%	In vitro recovery; stability confirmed by RAPD, REMAP, and ISSR analyses	[135,150,151,152,153,154]
*Rubus*, two cultivars	In vitro shoot meristems	Fast-freezing and PF	>1 h	10–86%	In vitro recovery	[141]
*Sorbus*, two cultivars	In vitro shoot meristems	Fast-freezing	>1 h	79–83%	In vitro recovery	[153]
*Rubus*, four mericlones	In vitro shoot meristems	Fast-freezing	>1 h	63–85%	In vitro recovery	[153]
*Syringa*, two cultivars	In vitro shoot meristems	Fast-freezing	Several weeks	83–84%	In vitro recovery	[145]

F—programmed freezing.

**Table 4:** Composition of collection of microalgae and cyanobacteria IPPAS as of 2023 ^1^.

Total Number of Strains	430
Identified to family level	27
Identified to genus level	403
Identified to species level	236
Total number of genera	91
Total number of species	106

^1^ The taxonomic position of the IPPAS strains is identified following the classification provided by Algaebase [171].

**Table 5:** The most notable and/or widely used strains of the IPPAS collection of microalgae and cyanobacteria.

Strain	Year Strain Isolated/Received by Collection	Characteristics
*Chlorella sorokiniana* IPPAS C-1	1961/1961	Unicellular coccoid green microalgae with robust growth characteristics; thermotolerant [187]; used in studies on endogenous regulation of photosynthesis and metabolism [188,189,190,191,192,193,194,195]; under stressed conditions, it accumulates mainly starch [191,192]; acts as a reference strain for biotechnological applications [175,188,189,196,197,198].
*Chlorella vulgaris* IPPAS C-2	n.a./1957	Unicellular coccoid green microalgae with robust growth characteristics; under stressed conditions, it accumulates mainly lipids [192]; acts as a reference strain for biotechnological applications [172,192,194,199,200]. The world’s first culture that was exposed to space flight conditions on the second spacecraft satellite [201].
*Neochlorella semenenkoi* Krivina, Temraleeva, Bobrovnikova and Sinetova IPPAS C-1210	2014/2014	Unicellular coccoid green microalgae with robust growth characteristics; thermotolerant, halotolerant, and alkaliphilic; accumulates lipids enriched with α-linolenic FA [202,203].
*Crocosphaera subtropica* IPPAS B-1603 Synonym: ‘*Cyanothece* sp.’ ATCC 51142	1992/2012	Unicellular marine nitrogen-fixing cyanobacterium; a model organism for studying circadian and ultradian rhythms [204]; possesses highly active extracellular α-class carbonic anhydrase [181].
*Cyanobacterium* sp. Rippka and Cohen-Bazire IPPAS B-1200	2013/2013	A unicellular alkaliphilic cyanobacterium with a wide temperature optimum (24–34 °C); contains many short saturated and monounsaturated C14 and C16 FAs, which can be used for biofuels [176]; draft genome sequenced [205]; possesses a unique desaturase, DesC, that nonspecifically introduces double bonds in C14, C16, and C18 FAs [206].
*Desertifilum tharense* IPPAS B-1220	2013/2013	A filamentous thermophilic cyanobacterium with a wide temperature optimum (28–40 °C); contains many 16:2Δ7,10 FAs rarely found in cyanobacteria [207]; draft genome sequence available [208].
*Sodalinema gerasimenkoae* IPPAS B-353	1996/1996	A filamentous haloalkaliphilic cyanobacterium; reference strain for the species [209]; a model organism for studying CO_2_ -concentrating mechanisms [180].
*Synechocystis* sp. IPPAS B-1400	1968/2005	Strain PCC 6803 GT-L, a model cyanobacterium for studying mechanisms of intracellular regulation and stress responses of photosynthetic cells [182,183,184,185,186]; wild type for many regulatory mutants; was used for the development of a new method for the growth characterization and optimization [210] and for studying quantitative growth properties and resource allocation [211].
*Vischeria punctata* IPPAS H-242	1941/1958	A unicellular coccoid eustigmatophycean microalgae; rich in eicosapentaenoic FAs [202,212].

## Data Availability

The lists of the collection strains are available in the online catalogs at http://ippras.ru, accessed on 17 May 2023.

## Appendix A

**Table A1:** Summary information about in vitro and cryobank collections at IPPRAS.

Collection	IPPAS Collection of Microalgae and Cyanobacteria	In Vitro Collection of Potato Plants	All-Russian Collection of Plant Cell Cultures	Adventitious Root Collection *	Cryobank
**Type of material conserved**	Microalgae and cyanobacteria (wild types and mutants)	In vitro plants	Undifferentiated cell cultures (calli, cell suspensions)	Adventitious roots	Shoot apices of in vitro plants, cell cultures, in vitro cultured protocorms, seeds
**Year collection established**	1958	2006	1965	2020	1977
**Age of the oldest samples**	Over 80 years	17 years	64 years	2 years	46 years
**Total number of taxa/lines in the collection**	91 genera/106 species/430 strains	*Solanum tuberosum*/202 lines	15 families/51 species/117 cell strains	7 families/12 species/12 strains	74 families/457 species
**Conservation methods**	1. In vitro with periodic subcultures (active collection) 2. Cold storage at −80 °C	In vitro with periodic subcultures (active collection)	1. In vitro with periodic subcultures (active collection) 2. Cryopreservation at −196 °C (old and the most productive cell strains)	In vitro with periodic subcultures (active collection)	Storage in LN at −196 °C (long-term conservation)
**Material acquisition sources**	1. External (research collections in and outside Russia) 2. Expeditions and strain isolation	1. External (research institutes in Russia and Belarus) 2. In-house developed transgenic lines	1. IPPRAS 2. Cell lines received from external users for maintenance (patent) purposes	IPPRAS	1. In-house developed clones and cell lines 2. Agricultural research institutes 3. Botanic gardens
**Fundamental research**	Mechanisms of intracellular regulation and stress responses in photosynthetic cells	Molecular mechanisms of disease resistance in potato	Plant cell physiology and biochemistry, growth regulation, and secondary metabolism	Secondary metabolism and its regulation in adventitious roots	Mechanisms of plant cold- and cryo-tolerance development, cryopreservation-induced injuries
**Applied research**	Production of biofuels, pigments, feed, and food additives	1. Development and optimization of potato transformation protocols 2. Development of disease-resistant potato lines	Production of vegetative biomass and bioactive compounds for food, cosmetics, and pharmacology	Biotechnological production of plant bioactive compounds	Viability of plant material during long-term storage, evaluation of material integrity and performance after cryopreservation
**Services to external users**	1. Strain deposit (maintenance) for the patent purpose 2. Taxonomic identification 3. Evaluation of the biotechnological potential of the strains 4. Strain distribution with request to research and commercial organizations	Development of transgenic potato lines carrying specific genes	1. Cell culture induction, optimization, and evaluation 2. Passport development for new cell lines 3. Cell culture deposit (maintenance) for the patent purpose	Currently not available	Long-term conservation of botanical, agricultural, and biotechnological collections

* The collection of hairy root culture was comprehensively covered earlier [17].

**Table A2:** Standard growth evaluation parameters usually estimated for plant cell cultures.

Parameter	Formula
Growth index, I	I = (X_i_ − X_0_)/X_0_
where X_0_ and X_i_ are initial dry weight and dry weight at the end of the exponential growth phase, respectively (g·L^−1^).	
Specific growth rate, μ (d^−1^)	µ = (1nX_i_ − lnX_0_)/∆t
where X_0_ and X_i_ are initial dry weight and dry weight at the end of the exponential growth phase, respectively (g·L^−1^); ∆t is the duration of the exponential growth phase specific for each culture.	
Economic coefficient, Y	Y = (X_max_ − X_0_)/S_0_
where X_0_ and X_max_ are initial dry weight and maximum dry weight (g·L^−1^), and S_0_ is the initial sucrose concentration in culture medium (g·L^−1^).	
Productivity, P (g/(L d))	P = (X_max_ − X_0_)/(t_i_ − t_0_)
where X_0_ and X_i_ are initial dry weight and maximum dry weight (g·L^−1^), and t_0_ and t_i_ are the time points at inoculation (0 days) and at maximum dry weight accumulation, respectively.	

**Table A3:** The current composition of adventitious root culture collection at IPPRAS.

Taxon		Culture Medium Type	
Family	Species	Agar-Solidified	Liquid
Araliaceae	*Polyscias filicifolia* L.H. Bailey	+	+
	*Polyscias scutellaria* (Burm.f.) Fosberg	+	+
	*Polyscias balfouriana* L. H. Bailey	+	+
Fabaceae (Leguminosae)	*Sutherlandia frutescens* (L.) W. T. Aiton	+	−
	*Maackia amurensis* Rupr.	+	+
Lamiaceae	*Ziziphora pamiroalaica* Juz. ex Nevski	+	−
	*Ziziphora interrupta* Juz.	+	−
Plantaginaceae	*Digitalis lanata* Ehrh.	+	+
	*Digitalis ciliata* Trautv.	+	−
Amaryllidaceae	*Pancratium maritimum* L.	+	+
Crassulaceae	*Kalanchoe laciniata* (L.) DC.	+	+
Betulaceae	*Corylus avellane* L.	+	+

Adventitious root cultures are maintained by periodic subcultures in the climate control chamber at 25 °C and relative air humidity of 60–70% in darkness. The majority of stock cultures are maintained on agar-solidified medium in Petri plates. Several lines of adventitious roots are grown in liquid medium in 250 mL flasks (40–50 mL of medium in the flask) on an orbital shaker (95 rpm).

**Table A4:** Countries of origin of the strains in the collection of microalgae and cyanobacteria IPPAS.

Country of Origin	Number of Strains
Russia	113
USA	20
Vietnam	7
Mongolia	7
Kazakhstan	7
Switzerland	6
Czech Republic	6
Germany	4
Uzbekistan	3
Indonesia	2
United Kingdom	2
Japan	2
Moldova	2
Italy	2
Azerbaijan	2
Kenya	2
Republic of Chad	2
Ethiopia	1
Netherlands	1
Slovakia	1
Western Sahara	1
Australia	1
Belgium	1
Tajikistan	1
Peru	1
Tanzania	1
Republic of Palau	1
Turkmenistan	1
Georgia	1
India	1
Tajikistan	1
Ukraine	1
France	1
n/a	225

**Table A5:** Main sources of the strains in the collection of microalgae and cyanobacteria IPPAS.

Collection/Institution, Country, Town	City, Country	Number of Strains (% in the Collection)	Year of the Deposition
Working collection of Dmitry Los and his group, Institute of plant physiology of Russian Academy of Sciences, http://cellreg.org/http-en-cellreg-org/, accessed on 20 April 2023	Moscow, Russia	91 (21%)	2005–2022
Collection staff expeditions and strain isolation, Institute of plant physiology of Russian Academy of Sciences	Moscow, Russia	56 (13%)	1968, 1983–1991, 2014–2021
Collection of *Chlamydomonas reinhardtii* photosynthetic mutants of Vladimir Ladygin, Institute of Basic Biological Problems, Russian Academy of Sciences	Pushchino, Russia	54 (13%)	2009
CALU, Collection of Algae St. Petersburg (Leningrad) State University, Centre for Culture Collection of Microorganisms, St. Petersburg State University, https://researchpark.spbu.ru/en/collection-ccem-eng/1930-ccem-kollekciya-calu-eng, accessed on 20 April 2023	St. Petersburg, Russia	16 (4%)	1969, 1987–1993, 2011
Pringsheim’s (Prat’s) algal collection, Charles University (currently does not exist)	Prague, Czech Republic	16 (4%)	1957–1964
Working collections of Dr. Lyudmila Gerasimenko and Dr. Olga Samylina, Winogradsky Institute of Microbiology, Federal Research Center “Biotechnology”, Russian Academy of Sciences	Moscow, Russia	14 (3%)	1979, 1996, 1998, 2019
Working collection of Olga Sentsova, Biology Faculty, Moscow State University	Moscow, Russia	12 (3%)	1990
LABIK, Collection of Algal Cultures in Laboratory of Algology, Komarov Botanical Institute of Russian Academy of Sciences	St. Petersburg, Russia	11 (3%)	1989, 1993
CCALA, Culture collection of Autotrophic Organisms (CCALA) of the Institute of Botany of the Czech Academy of Sciences, https://ccala.butbn.cas.cz/en, accessed on 20 April 2023	Třeboň, Czech Republic	10 (2%)	1964, 1988–1989
NAMSU, collection of biotechnologically relevant microalgae of Faculty of Biology, Moscow State University	Moscow, Russia	10 (2%)	2013–2014, 2017–2022
Working collection of Dr. Hiroshi Nakamura, The Institute of Algological Research (currently does not exist)	Japan	8 (2%)	1960
ACSSI, Algal Collection of Soil Science Institute, Institute of physicochemical and biological problems in soil science of the Russian Academy of Sciences	Pushchino, Russia	8 (2%)	2019–2021
Working collection of Ziyadin Ramazanov, K. A. Timiryazev Institute of plant physiology Russian Academy of Sciences	Moscow, Russia	7 (2%)	1980, 1984, 1996
Working collections from Institute of plant physiology (now Institute of plant physiology and genetics) of Bulgarian Academy of Sciences	Sofia, Bulgaria	6 (1%)	1970, 1978–1979, 1986
Working collection of Prof. Bolatkhan Zayadan group, Faculty of Biology and Biotechnology of the Al-Farabi Kazakh National University	Almaty, Kazakhstan	6 (1%)	2013
IAM, Algal Collection of Institute of Applied Microbiology, the University of Tokyo, Japan (does not exist know—moved to NIES, Microbial Culture Collection at the National Institute for Environmental Studies, https://mcc.nies.go.jp/07imformation_IAM_e.html, accessed on 20 April 2023)	Tsukuba, Japan	5 (1%)	1981, 1984, 1993
SAG, Culture Collection of Algae at Göttingen University, https://uni-goettingen.de/en/45175.html, accessed on 20 April 2023	Göttingen, Germany	5 (1%)	1983, 1990
Working collections of the researchers of the Vavilov Institute of General Genetics, Russian Academy of Sciences	Moscow, Russia	5 (1%)	1961, 1967
